# Identifying and assessing the capacity and experience of trial sites in low- and middle-income countries for high-quality randomised drug trials in maternal and perinatal health

**DOI:** 10.1136/bmjgh-2024-018063

**Published:** 2025-07-27

**Authors:** Maureen Makama, Annie RA McDougall, Anna Shalit, Ben Sanderson, Tahlia Guneratne, Kate Mills, Jenny Cao, Shivaprasad S Goudar, Pisake Lumbiganon, Anne Ammerdorffer, Jennifer A Scott, Lindsay Keir, Ahmet Metin Gülmezoglu, Joshua Peter Vogel

**Affiliations:** 1 Women's, Children's and Adolescents’ Health Program, Burnet Institute, Melbourne, Victoria, Australia; 2 School of Public Health and Preventive Medicine, Monash University, Melbourne, Victoria, Australia; 3 Monash Institute of Pharmaceutical Sciences, Monash University, Melbourne, Victoria, Australia; 4 Women's and Children’s Health Research Unit, KLE University, Belgaum, Karnataka, India; 5 Obstetrics and Gynaecology, Khon Kaen University Faculty of Medicine, Khon Kaen, Thailand; 6 Concept Foundation, Geneva, Switzerland; 7 Impact Global Health, London, UK

**Keywords:** Epidemiology, Global Health, Health services research, Obstetrics, Public Health

## Abstract

Existing international consortia for drug trials in maternal and perinatal health have focused largely on pragmatic trials using off-label medicines. This study aimed to identify and assess the capacity and experience of sites in low- and middle-income countries (LMICs) for conducting trials for regulatory approval of medicines for pregnancy-related conditions. We systematically reviewed site assessment checklists across any disease area to develop a maternal trial site assessment checklist. The checklist was pretested, revised and used to collect data from trial sites in LMICs. Sites were systematically identified from a scoping review of maternal trials conducted in LMICs, known networks and snowball searching. Data reported by sites were verified against publicly accessible sources (clinical trial registries and published articles). We contacted 106 sites in 30 countries, of which 49 (46.2%) sites in 21 countries completed the checklist. Sites were from five regions—Sub-Saharan Africa (37), South Asia (6), Latin America and the Caribbean (4), Middle East and North Africa (1) and East Asia and the Pacific (1). More than 70% of responding sites had the requisite physical infrastructure, clinical and research staff, ethics, participant recruitment and data management services to conduct randomised trials. Respondents collectively identified 52 completed, ongoing or planned maternal trials across their sites. Of these 52 trials, 16 (30.8%) were Good Clinical Practice-compliant, 22 (42.3%) were phase III and one was a regulatory trial. 14 trials were conducted by a collaborative group established mostly for a specific trial or a small group of related trials. Only two of these groups were pre-established trial networks. While there is some capacity to conduct high-quality maternal drug trials in LMICs, effective research collaborations are needed to further strengthen and expand this capacity. Establishing a sustainable LMIC-based trial network will accelerate the development and testing of novel drugs to improve maternal and newborn health outcomes in these regions.

**WHAT IS ALREADY KNOWN ON THIS TOPIC**

- Women living in low- and middle-income countries (LMICs) experience the greatest burden of pregnancy-related complications, yet most of the clinical trials involving pregnant women are conducted in high-income countries.
- Barriers to conducting clinical trials in LMICs such as limited research funding and skilled personnel can be overcome through establishing strong collaborative clinical trial networks.

**WHAT THIS STUDY ADDS**

- We developed the first maternal and perinatal health trial site assessment checklist.
- We used the checklist to identify trial sites capable of conducting high-quality trials of maternal medicines in LMICs.
- This study provides insights into the types and quality of maternal trials, as well as the sponsors and funders of these trials in LMICs.

**HOW THIS STUDY MIGHT AFFECT RESEARCH, PRACTICE OR POLICY**

- This site assessment study is the first step towards establishing a maternal trial network to consolidate research efforts and facilitate collaboration among trialists in LMICs.
- The checklist we created can be used to identify more sites and expand the database of sites capable of conducting maternal and perinatal health trials in LMICs.
- It is a valuable resource for researchers, trial sponsors and funders to guide site selection for future multicountry and/or multisite maternal and perinatal health trials.

## Introduction

 Complications of pregnancy are the leading causes of maternal and newborn deaths.^1^ In 2020, an estimated 2 87 000 women died from preventable pregnancy-related complications, 95% of which occurred in low- and lower-middle-income countries (LMICs).^1 2^ Many of these pregnancy complications can also cause long-term health consequences for women and children, such as cardiovascular diseases in women who developed pre-eclampsia or cognitive and neurological disabilities in children born preterm.^1 3^

The lack of research and development in medicines for pregnancy-specific conditions is a major barrier to progress in maternal and newborn health outcomes.^4^,^6^ In the past 30 years, only four drugs have been specifically developed and reached markets for pregnancy-related complications—brexanolone, zuralonone (both for postpartum depression), atosiban (tocolytic) and heat-stable carbetocin (uterotonic).^7^,^10^ This situation is often described as the ‘maternal drug drought’. Developing new medicines for pregnant and postpartum women faces several barriers.^48 11^,^14^ One such barrier is the historical exclusion of pregnant women from clinical trials, due to concerns about fetal exposure and safety and the associated implications for ethical approvals, safety monitoring and indemnity.^15^,^17^ Complex regulatory requirements for including pregnant women in clinical trials also create a barrier to their participation.^18^ Pharmaceutical companies are concerned about the risk of liability from research involving pregnant women and the associated financial and reputational costs.^19^ Thus, drug developers focus on chronic health conditions in non-pregnant adults for which there are larger markets and on whom it is less risky to test new products on.^5 7 19^ Consequently, using repurposed medicines is common practice in maternal health.

While women living in LMICs experience the greatest burden of pregnancy-related complications, most of the clinical trials involving pregnant women are conducted in high-income countries (HICs).^2 20^ A 2018 study analysed the distribution of sites conducting clinical trials between 2006 and 2012 and found that 83% of the pregnancy and childbirth trials were conducted in HICs.^20^ There remain significant barriers to the conduct of clinical trials in LMICs, including a lack of research funding, skilled personnel and limited mentorship, as well as regulatory and administrative challenges.^21 22^ Identifying sites capable of conducting Good Clinical Practice (GCP)-compliant regulatory trials is a first step towards addressing the barriers to conducting clinical trials, as well as understanding the challenges to accelerating the development and introduction of new products for pregnancy-specific conditions, in these regions.

The aim of this study was to identify and assess the capacity and experience of clinical trial sites for high-quality, phase III regulatory trials of novel medicines for pregnant and postpartum women living in LMICs. Regulatory trials are trials performed to produce data for government or regulatory agency’s approval of a product. We defined capacity as the ability of trial sites to perform the requisite functions sustainably and continually over time for the effective, ethical and safe conduct of maternal and perinatal health trials. We focused on clinical trial experience as a tangible outcome to measure capacity to conduct maternal and perinatal health trials.

## Methods

This study involved three key steps. First, a systematic review was conducted to inform the development of a site assessment checklist. Then, systematic mapping to identify existing LMIC-based sites with potential to support maternal and perinatal health trials was performed. The site assessment checklist was pretested in four of these sites and subsequently revised. Finally, using the revised checklist, data were collected from the identified sites.

### Systematic review and checklist development

We developed a checklist to assess the capacity of clinical trial sites for conducting regulatory trials in maternal and perinatal health using methods adapted from Salami *et al*.^23^ The checklist was developed following a systematic review (online supplemental appendix 1) of available trial site readiness checklists, which included all checklists with usable tools for assessing sites for human clinical trials, following Preferred Reporting Items for Systematic Reviews and Meta-Analyses reporting guidelines.^24^

#### Data extraction

The following data were extracted from included checklists: country of use, discipline or condition it was developed for, checklist development method (validated or not), the type of trials it pertained to: (regulatory or GCP-compliant trials) and how the checklist was intended to be used (remote interview or inperson assessment of site, at time of interview or retrospectively). Information about the contents of each checklist including the broad domains and any scoring system used was extracted.

The review team consolidated the extracted assessment items to create an initial draft checklist using items considered relevant to interventional trials in maternal and perinatal health. The initial draft was revised following consultations with the research team, experienced trialists, trial monitors and clinician-researchers based in LMICs.

### Identifying sites, checklist pretesting and revision

LMICs were defined using World Bank income level classifications.^25^ The 2024 classification identifies 134 countries as LMICs. A systematic approach was used to identify sites with potential to support maternal and perinatal health trials (defined as a facility or hospital where clinical trials are being conducted). This included:

1. Systematic selection from a scoping review of maternal and perinatal health trials in LMICs, published between 2010 to 2019.^26^ Sites conducting trials with larger sample sizes (≥1000) and reporting GCP-compliance and/or funding were selected; sites with potential integrity issues (retracted studies due to unreliable or ethical concerns), sites not active in the last 5 years and sites with apparent low potential (ie, only small or non-interventional trials conducted) were deprioritised.
2. Identification of sites from known clinical trial networks, for example, the World Maternal Antifibrinolytic (WOMAN) trial and WHO Antenatal CorticosTeroids for Improving Outcomes in Preterm Newborns (ACTION-I) trial.
3. Snowball sampling techniques through known contacts of trial sites.

Informed consent was provided by all participants before completing the checklist. Participants were informed that the information they provided would be made publicly available and consent was implied by completing the checklist.

#### Patient and public involvement

As an assessment of clinical trial site capacity, this study did not involve any patients or the public in its design or conduct.

#### Checklist pretest

The draft checklist (online supplemental appendix 2) was entered into Qualtrics, an online data collection platform.^27^ We selected and contacted seven LMIC-based sites via email to pretest the draft checklist. Sites were selected to provide geographical diversity. Semistructured interviews were scheduled over Microsoft Teams video calls with the participants in June and July 2023. The interview followed a predeveloped, semistructured interview guide (online supplemental appendix 3). Participants were sent a link to complete the online checklist before the interview but were also allowed to complete the checklist during the interview. During the interview, participants were asked to provide feedback on the structure of the checklist including ease of completion, its content or any other feedback. The checklist was revised (online supplemental appendix 4) based on the feedback received from the pretest interviews and used for data collection.

### Data collection from sites

A spreadsheet of existing sites with potential to support maternal and perinatal health trials was created, including the names and locations of the sites and the names and email addresses of the primary contact persons. All sites were contacted via email. A further two reminders were sent to those who did not respond, at 2-week intervals. Clarifications were provided via email to participants who required further details of the study. The revised checklist (online supplemental appendix 4) was available via Qualtrics27 and the link was shared with those who agreed to participate. The checklist was available only in English. Participants were also provided with the PDF version of the checklist (online supplemental appendix 4) to facilitate information gathering before the online checklist completion. A database of the characteristics of the sites was prepared as a Microsoft Excel spreadsheet.

As part of data collection, respondents were asked to list all maternal and perinatal health trials completed at the respective sites in the past 5 years, currently ongoing trials and trials planned for the next 2 years. They were also asked to list trials they completed in the past 5 years on topics outside of maternal and perinatal health. The trials reported by study sites were verified by checking them against publicly available records including published articles and clinical trial registries. The verified information included the trial status (completed, ongoing or planned), trial phase and whether the trial was GCP compliant, for regulatory approval of a drug, prospectively registered, randomised, placebo-controlled, multisite, multicountry or conducted as part of a trial network. We also verified information about funding, trial sponsors and any external partner institutes. Data at the site and trial levels were summarised descriptively using frequencies and percentages and presented in tables. Relationships between trial characteristics were explored using Spearman’s rank correlation analysis in Stata V.18.0.^28^

## Results

### Systematic review and checklist development

22 checklists (reporting 1752 assessment items) were included in the systematic review. Six checklists did not include a publication date; the remainder were published between 2013 and 2021. Only three checklists reported their method of development—all three had been validated via pilot testing in 9 to 12 sites. Some checklists were developed for use in specific countries or regions (one for Africa, one for Vietnam and two for the USA), while the others did not specify any setting (or were intended to be used in diverse settings). None of the included checklists were developed for use by a specific trial site.

Four checklists were product-specific (two for vaccines, one for oral medications and one for continuous positive airway pressure devices). None of the identified checklists were specifically for maternal or perinatal health trials. One checklist was specifically for ‘early-phase’ clinical trials, although it could be adapted for trials in any phase. Five checklists specifically mentioned being for assessing GCP compliance, and six were related to the conduct of regulatory trials.

Based on the assessment items identified in these 22 checklists, we developed the initial version of a maternal and perinatal health trial site checklist. The domains included in this checklist were: identifying information, research experience at the site, ethical and regulatory aspects, site characteristics, site clinical and research infrastructure, site staffing, site recruitment and data management.

### Identifying sites, checklist pretest and revision

Of the seven sites contacted to pretest the checklist, four sites (India, Nigeria, Papua New Guinea and Thailand) completed the pretest interviews. Participants considered the checklist easy to complete and suggested some changes. Based on feedback from the pretest interviews, the changes made to the checklist (resulting in version 2) included: defining terms such as ‘clinical investigator’; removing repetitions in questions regarding the contact details of investigators; clarifying that the checklist was to be completed per site; addition of questions about whether trials were regulatory or not and improving the overall flow and readability of the checklist by using ‘display’ and ‘skip’ logic functions in Qualtrics.^27^

### Data collection from sites

Of 115 sites contacted after the pretest, 45 sites responded and completed the checklist between November 2023 and March 2024, for a total of 49 sites included in the final database (including 4 pretested sites).

#### Site level information

**Figure 1:**
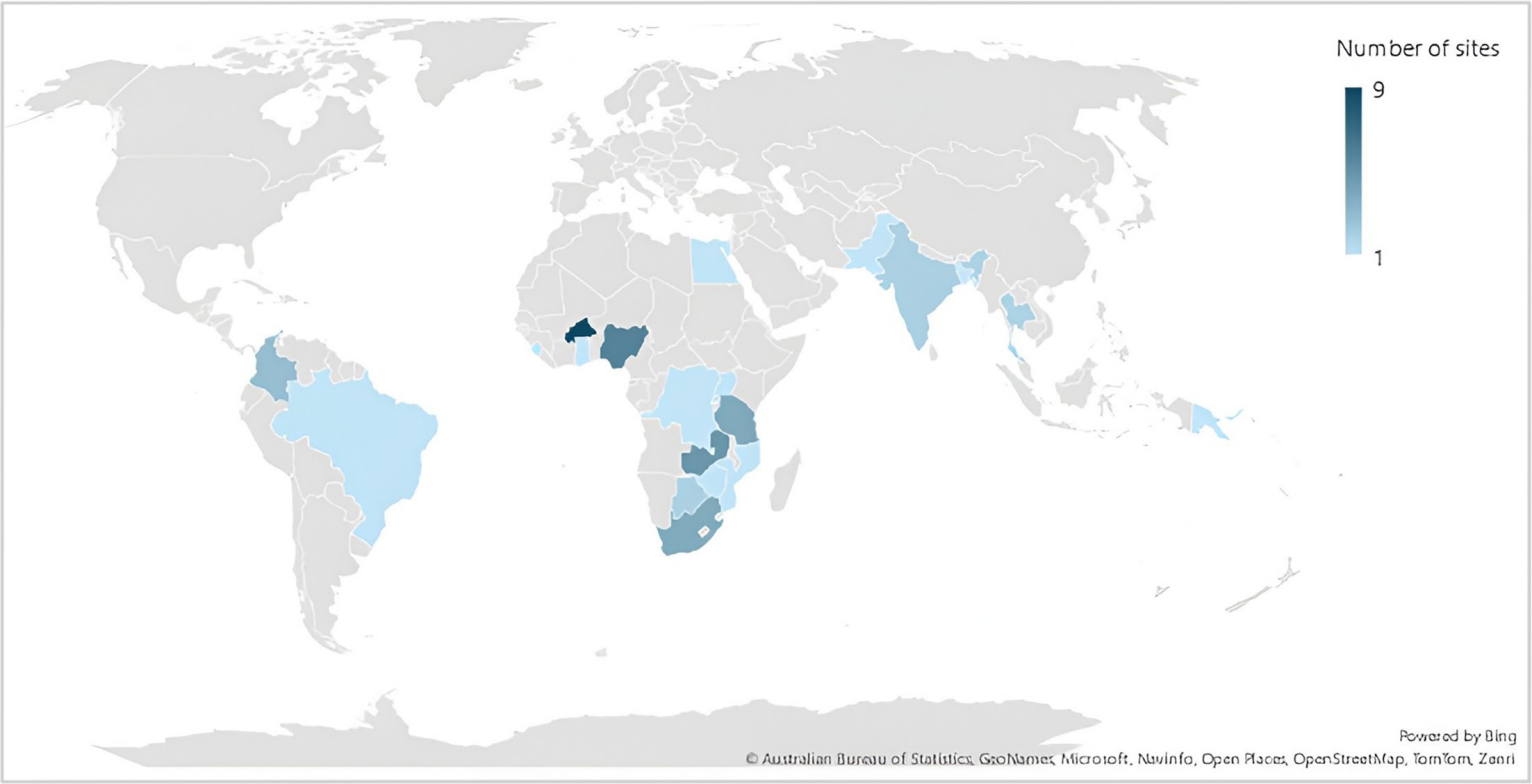
Number of study sites per country.

Responding sites were from 21 countries in five geographical regions. Most were in Sub-Saharan Africa (37 sites, 75.5%), six sites in South Asia (12.2%), four in Latin America and the Caribbean (8.2%), one (2%) in East Asia and Pacific and one (2%) in the Middle East and North Africa (table 1, figure 1). Of the 49 sites, 10 were noted to be research centres or institutes managing trials across multiple hospitals. Of which only one reported site details for all eight hospitals it managed.

**Table 1:** Number of study sites per World Bank region, according to the 2024 World Bank classification of countries by income level^23^

Region	Number of sites (%)
Sub-Saharan Africa	37 (75.5)
South Asia	6 (12.2)
Latin America and the Caribbean	4 (8.2)
East Asia and the Pacific	1 (2.0)
Middle East and North Africa	1 (2.0)
Total	49 (100.0)

### Previous, ongoing and planned trials reported by sites

Sites reported a total of 109 studies either completed in the last 5 years, ongoing or planned in the next 2 years on maternal and perinatal health topics. Of these, 57 were excluded following verification—39 were not randomised trials, nine were studies in children and nine were reported as planned trials yet could not be verified from publicly available information such as trial registries. This verification process resulted in 52 unique maternal and perinatal health trials.

#### Characteristics of the maternal and perinatal health trials

**Figure 2:**
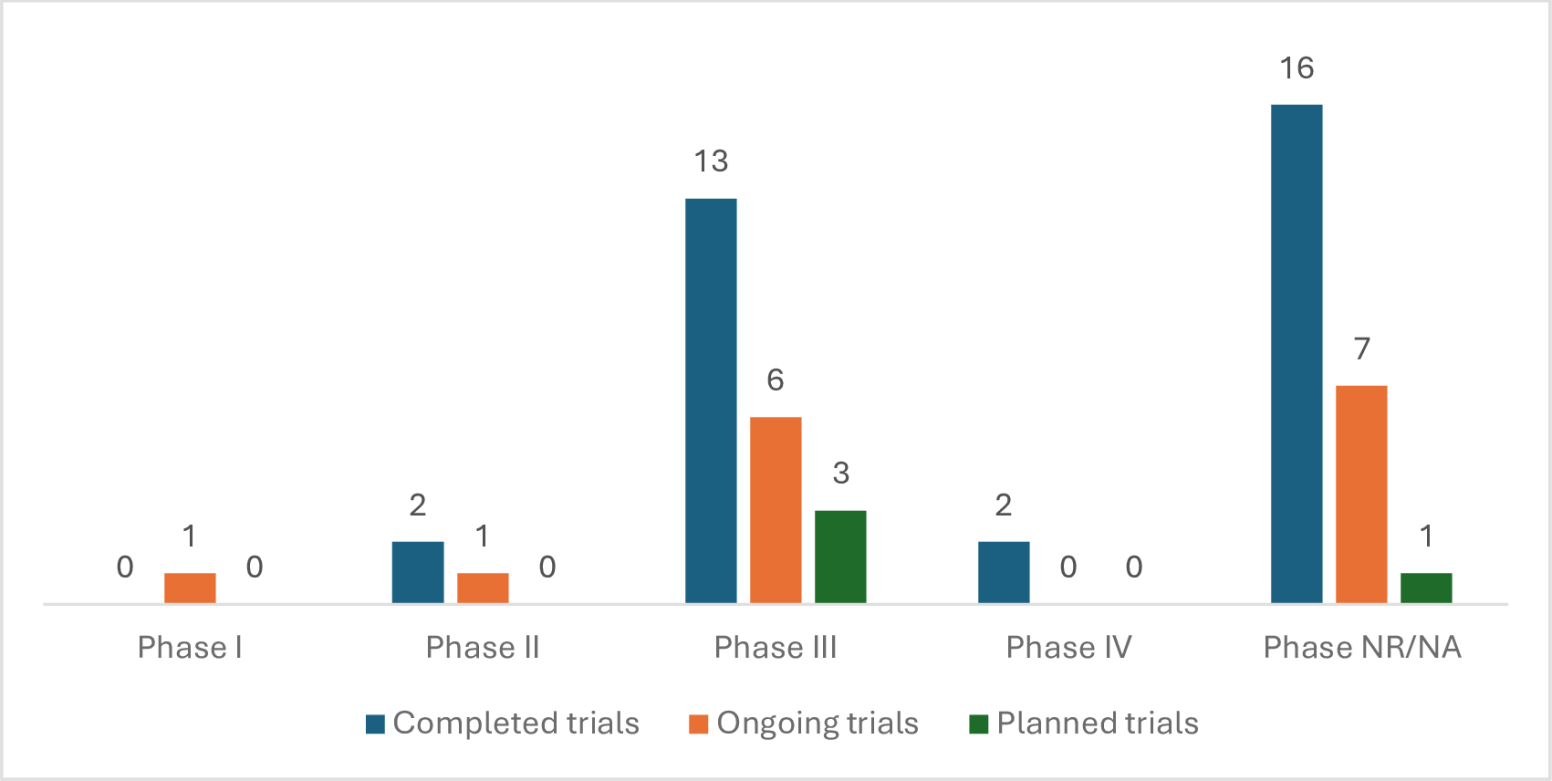
Phase of completed, ongoing and planned maternal and perinatal health trials (n=52 trials). NR/NA, not reported or not applicable (ie, for interventions other than drugs and vaccines).

Of the 52 maternal and perinatal health trials, 33 (63.5%) were completed, 15 (28.8%) were ongoing and four (7.7%) were planned (figure 2). 20 randomised controlled trials (RCTs) (38.5% of 52 trials) were placebo-controlled; 31 RCTs (59.6% of 52 trials) were not placebo-controlled and one RCT (1.9% of 52 trials) did not report whether it was placebo-controlled or not. Only 16 (30.8%) trials reported GCP compliance—the remaining 36 (69.2%) did not report GCP compliance status either in the published article or on a trial registry. The GCP-compliant trials were reported by 13 of the 49 (26.5%) sites. 22 of the 52 trials (42.3%) were phase III (figure 2). The types of interventions evaluated in the 52 maternal and perinatal health trials are shown in table 2. Most were drug trials (33 trials, 63.5%) (micronutrient supplements and vitamins were classified under drug trials). Up to 17.3% of trials were testing other interventions such as procedures, a checklist or guide or different combinations of a drug, a behavioural intervention, a device or a procedure. We identified only one regulatory trial—the Carbetocin Hemorrhage Prevention trial.^29^ Of the drug trials, five were trialling drugs not previously recommended by WHO for pregnancy-related complications. There were two metformin trials—Preeclampsia Intervention 2 (PI2) (phase II) and PI3 trials (phase III); one azithromycin trial (phase III)— Azithromycin-Prevention in Labor Use Study (A-PLUS) trial; one tranexamic acid trial (phase III)— World Maternal Antifibrinolytic (WOMAN) trial and one esomeprazole trial (phase II)—Esomeprazole for women at high risk of pre-eclampsia (ESPRESSO) trial.

**Table 2:** Types of interventions in the reported trials

Type of intervention	Number of trials n (%)
Drug trials	33 (63.5)
Behavioural trials	2 (3.8)
Device	3 (5.8)
Diagnostics	2 (3.8)
Vaccine trials	2 (3.8)
Health systems strengthening	1 (1.9)
Other	9 (17.3)
Total	52 (100)

The device trials tested a vacuum device for postpartum haemorrhage (Novel Vacuum-Induced Hemorrhage Control for Postpartum Hemorrhage (NOVIC) trial), postplacental placement of intrauterine devices and using the CRADLE device for automated blood pressure monitoring. The vaccine trials tested a two-dose Ebola vaccine regimen and respiratory syncytial virus maternal non-adjuvanted vaccine. The diagnostic trials tested the screening of term pre-eclampsia with an soluble fms-like tyrosine kinase-1/ placental growth factor (sFlt-1/PlGF) ratio and a digital clinical decision support tool (PANDA) for antenatal care.

#### Maternal and perinatal health trial networks

Of the 52 trials, 25 were multicountry and 41 multisite. 14 trials were part of a collaborative group (nine groups identified, table 3). All the collaborative groups were multicountry, except IVON-PP which was established to conduct a trial across 20 sites in four states in Nigeria.^30^ Of the nine collaborative groups identified, only two were pre-established networks (an existing clinical trial network), while the others were set up for a specific trial or group of trials. One of the two pre-established networks, one (International Maternal Pediatric Adolescent AIDS Clinical Trials Network) had a focus on the prevention and treatment of HIV in pregnant and lactating women and children.^31^ The other (Global Network for Women’s and Children’s Health Research) focused on complications and conditions related specifically to maternal and perinatal health.^32^ Both networks are funded by the US National Institutes of Health.

**Table 3:** Networks of maternal and perinatal health trials

Trial networks	Number of trials identified*	Intervention/health issue addressed	Network structure	Countries
Intravenous versus oral iron for iron deficiency anaemia	1	Intravenous iron versus oral iron for moderate to severe postpartum anaemia	For a specific trial	Nigeria
WHO Antenatal CorticosTeroids for Improving Outcomes in preterm Newborns Trial Collaborators	2	Antenatal corticosteroids for preterm birth	For a specific group of trials	India, Kenya, Nigeria, Pakistan, Bangladesh
PYRAPREG Consortium	1	Pyronaridine-artesunate for the treatment of malaria	For a specific trial	Burkina Faso, Democratic Republic of Congo, Mali, Mozambique, The Gambia
International Maternal Paediatric Adolescent AIDS Clinical Trials Network	1	Dolutegravir-containing versus efavirenz-containing antiretroviral therapy regimens for treating HIV-1-infected pregnant women and their infants	Established network	Zimbabwe; South Africa; Uganda; Brazil; Botswana; Tanzania; Thailand; USA; India
Early detection of Postpartum Haemorrhage and treatment using the WHO MOTIVE 'first response' bundle (E-MOTIVE) trial network	1	Treatment bundle of early detection, massage, oxytocic, tranexamic acid, intravenous fluid and examination of genital tract for management of PPH	For a specific trial	Kenya, Tanzania, Nigeria, South Africa and Pakistan
MAMAH trial group	1	Dihydroartemisinin-piperaquine for intermittent preventive treatment of malaria in HIV-infected pregnant women	For a specific trial	Gabon, Mozambique
World Maternal Antifibrinolytic Trial Collaborators	2	Tranexamic acid for treatment of PPH	For a specific trial	Nigeria, Pakistan, Uganda, Kenya, Cameroon, Sudan, UK, Tanzania, Nepal, Zambia, Albania, Democratic Republic of Congo, Bangladesh, Ethiopia, Jamaica, Burkina Faso, Ghana, Papua New Guinea, Egypt, Colombia, Côte d’Ivoire
Global Network for Women’s and Children’s Health Research	4	Intravenous iron vs oral iron for reducing anaemia in postpartum women – PRIORITY trial; low dose aspirin for reducing the rate of preterm birth – ASPIRIN; Azithromycin for reducing maternal and neonatal infections – A-PLUS trial; maternal nutritional supplementation before conception on newborn size – Women First trial	Established network	India, Zambia, Guatemala, Pakistan, Democratic Republic of Congo, Bangladesh
WHO Carbetocin Hemorrhage Prevention trial group	1	Heat-stable carbetocin vs oxytocin for PPH after vaginal birth	For a specific trial	Argentina, Egypt, India, Kenya, Nigeria, Singapore, South Africa, Thailand, Uganda and the UK

* This refers to the number of trials reported by sites in this study, the specified networks may have conducted more trials that were not reported.

AIDS, Acquired Immunodeficiency Syndrome; E-MOTIVE, Early detection of Postpartum Haemorrhage and treatment using the WHO MOTIVE 'first response' bundle; HIV, Human Immunodeficiency Virus; MAMAH, Improving Maternal heAlth by Reducing Malaria in African HIV Women; MOTIVE, uterine Massage, Oxytocic drugs, Tranexamic acid, Intravenous fluids, Examination and Escalation; PPH, Postpartum haemorrhage; PYRAPREG, Efficacy and Safety of Pyronaridine-Artesunate (PYRAMAXÂ®) for the treatment of *Plasmodium falciparum* uncomplicated malaria in African pregnant women; WHO, World Health Organization.

#### Funding of the 52 maternal and perinatal health trials in LMICs

A third of the reported trials (19, 36.5%; online supplemental table S1) were funded by public government organisations such as the National Institute of Health, the European and Developing Countries Clinical Trials Partnership, the European Commission and the Australian National Health and Medical Research Council. About a quarter of these trials (13 trials; 25.0%) were funded by philanthropic organisations such as the Bill and Melinda Gates Foundation, Thrasher Research Fund, Wellcome Trust and The Geoff and Helen Handbury Foundation. Six trials (11.5%) were funded by non-governmental or intergovernmental organisations including the Pre-eclampsia Foundation, Mercy Perinatal Foundation, Unitaid and the WHO. Three trials (5.8%) were funded by private multinationals like Procter and Gamble, GlaxoSmithKline Biologicals and Merck Sharpe & Dohme. Two trials (3.8%) were funded by private small-medium scale enterprises including Holley Pharmacy and Hope Specialist Healthcare. No funding was reported for five trials (9.6%) (online supplemental table S1). Philanthropic organisations funded larger trials compared with the trials funded by public government organisations. The largest trial funded by a philanthropic organisation had a sample size of 210 132 participants (the E-MOTIVE trial) across four countries while the largest trial funded by a public government organisation had a sample size of 29 278 participants (the A-PLUS trial) across seven countries.

#### Trial sponsors

Large multicountry trials were generally sponsored by groups located outside of the trial site (eg, the University of North Carolina and the University of Oxford). 45 trials (86.5%) had an external partner institute, defined as a partnership with any organisation outside the primary organisation where the trial was being conducted. Where LMIC-based sponsors were reported (eg, the Zvitambo Institute for Maternal and Child Health Research, Zimbabwe and the College of Medicine University of Lagos, Nigeria) the trials were single-country trials.

#### Correlation analysis

Trial sample size was positively correlated with trials being multicountry, multisite and being part of a network (n=47 trials, Spearman’s rank correlation coefficients 0.5513, 0.4144, 0.2950, respectively). There was a positive correlation between multicountry trials and being part of a network (n=52 trials, Spearman’s rank correlation coefficients 0.4098).

#### Site care facilities and infrastructure

To assess the capacity of sites in LMICs that are conducting maternal and perinatal health trials, self-reported data on the capacity of all 49 sites were collected. Most sites (>70%) reported having requisite ethical and regulatory aspects; adequate site infrastructure, utilities and services; clinical and research staff coverage; recruitment support and data management services (online supplemental appendix 5).

## Discussion

This is the first study to develop a site assessment checklist specific to maternal and perinatal health trials and systematically identify and assess the capacity of trial sites in LMICs. Our site assessment checklist is consistent with existing assessment tools in non-maternal health fields as it was developed following systematic review of existing tools. Most of the identified sites reported having the necessary care facilities and infrastructure for conducting maternal and perinatal health trials. Only 16 of 52 maternal and perinatal health trials reported by sites met all GCP compliance requirements. 22 (42% of 52) trials were phase III, but only one was for regulatory approval of a drug (heat-stable carbetocin).^29^ Nine collaborative groups were identified, of which only two were pre-established networks.

GCP compliance is necessary to protect the rights, integrity and confidentiality of trial participants.^33^ It is therefore important that trialists not only comply with GCP standards but also report it when publishing trial findings. In this study, details of trials reported by sites were validated from published records. Although most sites reported having GCP standards in place, only 16 of the 52 maternal and perinatal health trials reported it in published records. This may reflect a lack of reporting, rather than a lack of GCP compliance. It is paramount that scientific journals ensure that trial authors include a statement on GCP compliance as part of clinical trial publication.

In this study, trials that were multicountry, multisite or part of a network were more likely to have larger sample sizes. Clinical trial networks are collaborative groups of clinicians and researchers working to enhance the efficiency and quality of clinical research, through well-designed multisite trials.^34^ Trials conducted by clinical trial networks are more likely to influence clinical guidelines due to their rigour, size and power and expedite the translation of evidence into practice.^34^ Furthermore, established clinical trial networks afford training and mentorship opportunities that could build the expertise of clinicians and researchers in LMICs.^35^ For many of the maternal and perinatal health trials we identified, a new collaborative group was set up each time a trial (or group of trials) was planned, which did not outlive the trial itself. Considering the time and resources required to set up and execute high-quality clinical trials successfully, this is a significant inefficiency. By comparison, sustaining clinical trial networks is also resource-intensive—requiring funding support between trials—but would be more efficient, allow sites to better accumulate expertise and build capacity.^34 36^

By way of example, the Clinical Trials Community Africa Network (CTCAN) seeks to enable increased, sustainable and coordinated clinical trials in Africa.^37^ CTCAN consolidates relevant subnetworks of clinical trial sites and laboratories capable of undertaking stringent regulatory authority-quality clinical research in Sub-Saharan Africa.^37 38^ However, CTCAN does not specifically focus on sites with trial capacity for pregnant and lactating women (although such sites are eligible). Nevertheless, their approach could help establish a clinical trial network specific to maternal and perinatal health and to engage other LMICs outside of Africa. Another example is the Global Network for Women’s and Children’s Health Research, which is a partnership dedicated to improving maternal and child health outcomes and building health research capacity in LMICs. However, they mainly conduct large-scale observational studies and pragmatic randomised trials using off-label or established medicines. Networks such as the CTCAN and the Global Network could be leveraged to establish a platform for several collaborative groups to connect to broaden collaboration and increase the sustainability and efficiency of clinical trials of novel maternal medicines in LMICs. An attractive and feasible model that could strengthen trial capacity in LMICs is establishing a hub-and-spoke model. This would include a central hub of experienced and capable sites and spokes of sites with limited experience and capacity.

Funding is a critical limiting factor to drug trials in LMICs. There is limited government funding for research institutions in LMICs due to resource constraints and a lack of prioritisation of research in government budgets.^39^ LMIC triallists often depend on international grant funding, with low chances of success.^40^ Strong collaboration, mentorship and resource sharing through clinical trial networks not only strengthens research capacity, but can also increase funding success.^34 35 41^ Several LMIC research sites are linked to HIC research leadership and funding, such as the National Institute of Health’s Global Network for Women’s and Children’s Health Research.^32^ The relationships and power dynamics between hub leadership and trial sites impact research funding and alignment with priority health issues in LMICs. For many LMICs, funding sources shape research priorities and these may not align with local research needs. Equitable research partnerships are necessary to bridge the capacity divide between LMICs and HICs, ensure that trials conducted in LMICs address local needs and enable LMIC-based researchers to lead research occurring in their countries.^40^

### Implication for practice and research

This site assessment study is a valuable resource for researchers, trial sponsors and funders to guide site selection for future multicountry and/or multisite maternal and perinatal health trials. The site assessment checklist we created can be used to identify more sites and expand the database of sites capable of conducting maternal and perinatal health trials in LMICs. This database could be used to inform the establishment of a maternal and perinatal health trial network which would facilitate collaboration among researchers in LMICs, thereby strengthening clinical trial research capacity in these regions. In this study, we focus on trial site capacity; however, the individual researcher capability is also critical to the conduct of high-quality clinical trials in LMICs and should be explored in future studies. Knowledge of the research capabilities of LMIC-based researchers is important for understanding current capacity needs and developing strategies to address them. One consideration for strengthening research capability is through doctoral and postdoctoral research positions and increasing the number of clinicians involved in research.^35^

### Strengths and limitations

This study has several strengths. First, we conducted a systematic review to inform checklist development, ensuring the rigour and robustness of the checklist and associated data. This is the first publicly available site assessment checklist specifically for interventional maternal and perinatal trials. Second, we pretested and refined the checklist, ensuring its reliability and ease of use. Third, we employed a systematic approach to the selection of sites, ensuring representation from five 2024 World Bank regions. Finally, we checked the trials reported against publicly available sources including clinical trial registries and published articles, thus improving the validity of the report.

A limitation of this study is the response rate (~46%) despite multiple contacts to study sites. In addition, the site assessment checklist is currently only available in English, which could have limited some sites from completing it. None of the sites contacted requested the checklist in a different language; however, our future research will include translation of the checklist into other languages (Spanish, French) to expand our database of sites. Furthermore, sites that responded are likely to be more motivated and engaged than those that did not. However, the primary aim of this study was to identify promising sites, and the most engaged are also likely to have the greatest capacity. Non-respondents are most likely to benefit from capacity building through the hub-and-spoke model. Finally, we relied on self-reports for site characteristics, which could have introduced some biases.

## Conclusion

Mapping the current state of maternal and perinatal health trials and the sites conducting these trials in LMICs is a first step to understanding how they can be optimised. Linking sites together, consolidating research efforts and establishing a network among existing small networks may strengthen research capacity and collaboration. However, further work is needed to establish such a network among the sites we identified. This will involve an operational model, organised training to strengthen capacity, collaboration and knowledge sharing and a shared set of priorities and activities over time. This will allow promising new interventions to be more rapidly advanced through the research pipeline in LMICs. Such a network would constitute a significant benefit to the global maternal and perinatal health community.

## Supplementary material

10.1136/bmjgh-2024-018063 online supplemental appendix 1

10.1136/bmjgh-2024-018063 online supplemental appendix 2

10.1136/bmjgh-2024-018063 online supplemental appendix 3

10.1136/bmjgh-2024-018063 online supplemental appendix 4

10.1136/bmjgh-2024-018063 online supplemental appendix 5

## Data Availability

All data relevant to the study are included in the article or uploaded as supplementary information.
