# Supplementary material for: Identifying and assessing the capacity and experience of trial sites in low- and middle-income countries for high-quality randomised drug trials in maternal and perinatal health
Source: BMJ Glob Health. 2025 Jul 27;10(7):e018063. doi: 10.1136/bmjgh-2024-018063 (PMC12306368; doi:10.1136/bmjgh-2024-018063)
Supplement: online supplemental appendix 1 [file bmjgh-10-7-s001.docx]

Table of Contents

**Search strategy and screeningAppendix 1**

**Draft checklistAppendix 2**

**Interview guideAppendix 3**

**Revised checklistAppendix 4**

**The current state of sites conducting maternal and perinatal health trials in LMICsAppendix 5**

#### Appendix 1. Search strategy and screening

#### Search strategy

The following databases: MEDLINE, Embase, Global Index Medicus and Global Health were searched for articles published from inception to 17 August 2022. The search strategy combined terms relating to trial sites and forms of assessment (Table 1). We also searched the grey literature on 18 August 2022 by combining Google search and searching through specific websites of interest. The Google search used the Boolean operator “AND” to combine the term “trial site” with the terms “framework OR checklist OR scorecard OR questionnaire OR tool”. The first 10 pages of results were extracted for screening. The websites of the following agencies were searched: The World Health Organization (WHO), The Global Health Network, Food and Drug Administration (FDA), National Institutes of Health (NIH), Therapeutic Goods Association (TGA), National Health and Medical Research Council (NHMRC), Medical Research Council (United Kingdom Research and Innovation), European Medicines Agency (EMA), SwissMedic, Bill & Melinda Gates Foundation, Welcome Trust, The National Institute for Health and Care Excellence (NICE) and The International Federation of Gynaecology and Obstetrics (FIGO).

Table 1. Database search strategy

| Databases | Search strategy |
| --- | --- |
| Medline, EMBASE, Global Health | ((Trial site* OR trial location*) AND (framework* OR frame-work* OR checklist* OR check-list* OR scorecard* OR score-card*)) OR (((site assessment OR site readiness OR site capacity OR site selection) OR (trial site*)) AND (framework* OR frame-work* OR checklist* OR check-list* OR scorecard* OR score-card*)) OR (site readiness AND trial*) OR (assess* adj4 trial site*) OR (capacit* adj4 trial site*) OR (readiness* adj site*) OR (trial site* adj4 select*) OR (site select* adj4 trial) |
| Global Index Medicus | ((Trial site OR trial location) AND (framework OR frame-work OR checklist OR check-list OR scorecard OR score-card)) OR (((site assessment OR site readiness OR site capacity OR site selection) OR (trial site*)) AND (framework* OR frame-work* OR checklist* OR check-list* OR scorecard* OR score-card*)) |

##### Screening

Citations from the databases were screened using Covidence while the grey literature searches were screened using a Microsoft Excel spreadsheet. All screening was done by two independent reviewers. Table 2 summarizes the eligibility criteria.

Table 2. Eligibility criteria of articles for checklist development

| **Inclusion criteria** | **Exclusion criteria** |
| --- | --- |
| Tool or checklist | Not a usable tool/ checklist/ scorecard/ template/ questionnaire |
| Used for assessing site readiness before a trial e.g., used to decide if a site will be included in a study | Not used for assessing site readiness e.g., used for monitoring of a site during a clinical trial, used to see if the site is ready to start an intervention (when the site has already been decided upon) |
| Used for assessing the actual trial site | Not used for assessing trial sites e.g., used for assessing trial feasibility |
| For use in clinical trials | Not used for clinical trials e.g., used for assessing site readiness for manufacturing of a medication |
| Human clinical trials | Non-human trials |
| Used for the development of healthcare products | Used for the development of products not for healthcare |
