# Supplementary material for: Identifying and assessing the capacity and experience of trial sites in low- and middle-income countries for high-quality randomised drug trials in maternal and perinatal health
Source: BMJ Glob Health. 2025 Jul 27;10(7):e018063. doi: 10.1136/bmjgh-2024-018063 (PMC12306368; doi:10.1136/bmjgh-2024-018063)
Supplement: online supplemental appendix 2 [file bmjgh-10-7-s002.pdf]

Accelerating Innovation for Mothers (AIM): Site Readiness Checklist for conduct of Regulatory Trials in Maternal and Perinatal Health

Version: 1.0

Purpose: A checklist that can be used to assess the capacity and readiness of study sites for conducting clinical research and development trials in maternal and perinatal health that meet regulatory requirements.

Scope: The scope of this checklist is for clinical trials on major causes of maternal and perinatal morbidity and mortality. For example, trials that evaluate novel medicines or devices for the prevention, detection or treatment of postpartum haemorrhage, hypertensive disorders of pregnancy, preterm labour and birth, peripartum infections, and other complications of pregnancy, labour, and childbirth.

Setting: The setting of interest for this checklist are formal healthcare facilities (including primary, secondary, and tertiary level care) that are providing antenatal, intrapartum and/or postnatal care in low- and middle-income countries (LMICs). If a site also has access to delivering community-level interventions, and capacity for follow up of infants and mothers long-term, that would be an advantage.

Use:

- This AIM checklist is to be used as an initial assessment and can be completed remotely. It is likely that further detailed assessment (including in-person visits) may be required to capture additional detail or verify collected data.
- The checklist should be completed per individual site/facility or for a hub if the site is coordinating studies at several different sites.
- Completion date should be noted and regular updates (e.g., every 5 years) should be contemplated.

Development: This checklist was developed using the following steps:

1) We conducted a systematic review of clinical trial checklists in the peer-reviewed and grey literature, across any disease area. This review identified 22 checklists, which reported a total of 1752 assessment items. None of the checklists were specific to maternal and perinatal health, and some focussed on specific diseases (such as Huntington's disease, Lassa fever, and tuberculosis). Six checklists were specifically related to the conduct of regulatory trials.
2) The review team assessed and consolidated all 1752 items and created an initial draft of the AIM checklist (v1.0) using items that were considered relevant to interventional trials in maternal and perinatal health.

3) The initial draft was revised following consultations with the broader AIM team, as well as experienced triallists, trial monitors and clinician-researchers based in LMICs.
4) The revised checklist was then used to collect data from a sample of study sites in low- and middle-income countries. These sites were identified from a scoping review of clinical trials in maternal and perinatal health conducted in LMICs between 2010 and 2019 (Eggleston et al., 2022) and previous or current multi-centre trials. Further revisions were made based on lessons learned during data collection.

Definitions:

- Good Clinical Practice (GCP): an international ethical and scientific quality standard for trials that involve the participation of human subjects.
- Trial: Indicates an interventional randomised study
- Phase I trial: "first-in-human" studies. Designed to test safety of treatment.
- Phase II trial: Designed to test for biological activity or effect of treatment.
- Phase III trial: Designed to test for value in clinical practice.
- Trial Sponsor: The organization, company, institution, or group, that oversees the clinical trial and collects and analyses the data.

CHECKLIST

1. Identifying information

1. Study site<br>*: any physical location where a trial is conducted.*	Name:<br><br>Address:<br><br>City:<br>State:<br>Province:<br>Country:
2. Principal Investigator (PI)<br>*: lead researcher responsible for the conduct of a trial at any study site.*<br>☐ n/a	Full name:<br>Work address:<br><br>Phone number:<br>Email address:<br>Affiliation/s:<br><br>List of completed trials:<br><br><br>List of ongoing trials:<br><br>Valid Good Clinical Practice (GCP) qualification:<br>☐ Yes ☐ No
3. Primary Contact<br>*: interviewee.*<br>☐ n/a	Full name:<br>Work address:<br><br>Phone number:<br>Email address:<br>Affiliation/s:<br><br>Valid GCP qualification: ☐ Yes ☐ No
4. Site Co-ordinator<br>*: person responsible for managing clinical activities.*<br>☐ n/a	Full name:<br>Official address:<br><br>Phone number:<br>Email address:<br>Affiliation/s:<br><br>Valid GCP qualification: ☐ Yes ☐ No
5. Clinical Investigator(s)	Full name:<br>Official address:

: person responsible for the conduct of a clinical trial at a trial site.<br>☐ n/a	Phone number:<br>Email address:<br>Affiliation/s:<br><br>Valid GCP qualification: ☐ Yes ☐ No
	Full name:<br>Official address:<br><br>Phone number:<br>Email address:<br>Affiliation/s:<br><br>Valid GCP qualification: ☐ Yes ☐ No
	Full name:<br>Official address:<br><br>Phone number:<br>Email address:<br>Affiliation/s:<br><br>Valid GCP qualification: ☐ Yes ☐ No
6. Is there a finance administration team at this site?<br>*: person(s) responsible for maintaining financial security of a trial site.*	☐ Yes ☐ No
List the contact details of the budget manager for this site.<br>*: person primarily responsible for maintaining financial security of a trial site.*	Full name:<br>Work address:<br><br>Phone number:<br>Email address:
7. Does the site have a secure bank account that allows for	☐ Yes ☐ No

international money transfer?<br>*: capacity for electronic transfer of funds by an international money transfer provider.*	
Date this checklist was completed	Date:

2. Research experience of the site

1. Are there any trials in maternal and perinatal health **currently ongoing** at this site?<br><br>If yes, provide:<br>- The full name and clinical trial registration number for each trial<br>- The intervention that was being evaluated (such as drug or device)<br>- Indicate whether the trial is GCP-compliant.<br>- Indicate whether the trial was a Phase I, II or III trial	☐ Yes ☐ No<br><br>Full name:<br>Reg. no.:<br>Intervention:<br>☐ Drug ☐ Medical device<br>☐ Biological ☐ Vaccine ☐ Surgical<br>☐ Other treatment<br>GCP compliant: ☐ Yes ☐ No<br>Phase: ☐ I ☐ II ☐ III ☐ IV
2. Were there any trials in maternal and perinatal health **completed** at this site <u>in the past five years</u>?<br><br>If yes, provide:<br>- The full name and clinical trial registration number for each trial<br>- The intervention that was being evaluated (such as drug or device)<br>- Indicate whether the trial was GCP-compliant.<br>- Indicate whether the trial was a Phase I, II or III trial	☐ Yes ☐ No<br><br>Full name:<br>Reg. no.:<br>Intervention:<br>☐ Drug ☐ Medical device<br>☐ Biological ☐ Vaccine ☐ Surgical<br>☐ Other treatment<br>GCP compliant: ☐ Yes ☐ No<br>Phase: ☐ I ☐ II ☐ III ☐ IV
3. Are there any trials in maternal and perinatal health **planned** (i.e., the protocol is currently being formally considered by an ethics committee) at this site <u>in the next two years</u>?<br><br>If yes, provide:<br>- The full name and clinical trial registration number for each trial<br>- The intervention that was being evaluated (such as drug or device)<br>- Indicate whether the trial was GCP-compliant.	☐ Yes ☐ No<br><br>Full name:<br>Reg. no.:<br>Intervention:<br>☐ Drug ☐ Medical device<br>☐ Biological ☐ Vaccine ☐ Surgical<br>☐ Other treatment<br>GCP compliant: ☐ Yes ☐ No

- Indicate whether the trial was a Phase I, II or III trial	Phase: ☐ I ☐ II ☐ III ☐ IV
4. Have there been any trials in women's health **completed** at this site <u>in the past five years</u>?<br><br>If yes, provide:<br>- The full name and clinical trial registration number for each trial<br>- The intervention that was being evaluated (such as drug or device)<br>- Indicate whether the trial was GCP-compliant.<br>- Indicate whether the trial was a Phase I, II or III trial	☐ Yes ☐ No<br><br>Full name:<br>Reg. no.:<br>Intervention:<br>☐ Drug ☐ Medical device<br>☐ Biological ☐ Vaccine ☐ Surgical<br>☐ Other treatment<br>GCP compliant: ☐ Yes ☐ No<br>Phase: ☐ I ☐ II ☐ III ☐ IV
5. Has this site previously been part of any international trial networks?<br><br>If yes, provide details (name of the network, trial involved in)	☐ Yes ☐ No
6. Has this site conducted any other GCP compliant trials outside of maternal, perinatal or women's health in the past 5 years?<br><br>If yes, provide:<br>- The full name and clinical trial registration number for each trial<br>- The intervention that was being evaluated (such as drug or device)<br>- Indicate whether the trial was GCP-compliant.<br>- Indicate whether the trial was a Phase I, II or III trial	☐ Yes ☐ No<br><br>Full name:<br>Reg. no.:<br>Intervention:<br>☐ Drug ☐ Medical device<br>☐ Biological ☐ Vaccine ☐ Surgical<br>☐ Other treatment<br>GCP compliant: ☐ Yes ☐ No<br>Phase: ☐ I ☐ II ☐ III ☐ IV

3. Ethical and regulatory aspects

1. Does the site have an ethics committee?<br>*: committee responsible for evaluating the ethical aspects and scientific validity of a proposed study (sometimes known as IRB). In some cases, there may be separate committees for ethics and scientific methods.*<br><br>If yes, how often does the ethics committee meet?	☐ Yes ☐ No
2. Does the site have internal guidelines (e.g., participant care guidelines) and/or Standard Operating Procedures (SOPs) related to the following aspects of clinical trial management:<br>i. Safety management (e.g., responding to adverse events (AEs) / serious adverse events (SAE))<br>ii. Participant recruitment<br>iii. Clinical trial procedures<br>iv. Training and quality management	 <br><br><br><br>☐ Yes ☐ No<br><br><br>☐ Yes ☐ No<br>☐ Yes ☐ No<br>☐ Yes ☐ No
3. In the past five years, has this site been required to stop recruiting for any trial due to a serious safety concern, such as by an ethics review committee or regulatory body?<br><br>If yes, please describe the circumstances	☐ Yes ☐ No
4. Is abortion allowed in the country?	☐ Yes, until 8 weeks<br>☐ Yes, until 12 weeks<br>☐ Yes, no legal gestational limit<br>☐ Yes, only for medical reasons<br>☐ No

4. Site characteristics

1. Location<br>(Tick all that apply)	☐ Urban<br>: *high population and density.*<br>☐ Rural<br>*: low population and density, centred on agricultural production.*<br>☐ Peri urban<br>: *mix of rural and urban characteristics.*
2. Level<br>(Tick all that apply)	☐ Tertiary<br>*: highly specialised medical care.*<br>☐ Secondary<br>: *specialist healthcare treatment and support.*<br>☐ Primary<br>*: first point of contact for healthcare.*<br>☐ Other: ______
3. Types of care provided at this site:<br>(Tick all that apply)	☐ Antenatal care<br>*: care during pregnancy.*<br>☐ Labour and childbirth care<br>*: care during childbirth.*<br>☐ Postnatal care<br>*: care following childbirth.*
4. If antenatal care is provided, how many antenatal visits are performed at this facility each month (on average)?<br>☐ n/a	
5. If labour and childbirth care is provided, how many births occur per month (on average, as per most recent year of data)?<br>☐ n/a<br><br>How many caesarean sections are performed (on average, as per most recent year of data)?<br>☐ n/a	
6. If postnatal care is provided, how many postnatal visits are performed at this facility each month (on average)?<br>☐ n/a	

7. Are maternal and newborn care services located within the same hospital?	☐ Yes ☐ No
8. Which of the following services are available at this hospital:	
i. Administration of parenteral antibiotics<br>*: administration of antibiotics by injection methods*	☐ Yes ☐ No
ii. Administration of uterotonic drugs (i.e., parenteral oxytocin, tranexamic acid)<br>*: administration of drugs designed to induce contraction of the uterus*	☐ Yes ☐ No
iii. Administration of magnesium sulfate for pre-eclampsia and eclampsia	☐ Yes ☐ No
iv. Manual removal of the placenta	☐ Yes ☐ No
v. Removal of retained products (e.g., manual vacuum extraction, dilation, and curettage)	☐ Yes ☐ No
vi. Assisted vaginal birth (e.g., vacuum extraction, forceps)	☐ Yes ☐ No
vii. Basic neonatal resuscitation (e.g., with bag and mask)	☐ Yes ☐ No
viii. Caesarean section	☐ Yes ☐ No
ix. Breech delivery, multiples	☐ Yes ☐ No
x. Surgery	☐ Yes ☐ No
xi. Safe abortion (medical and/or surgical)	☐ Yes ☐ No
5. Are women with obstetric complications (such as severe pre-eclampsia, peripartum sepsis, postpartum haemorrhage) treated at:	
i. This site	☐ Yes ☐ No
ii. Referred to a higher-level facility	☐ Yes ☐ No

5. Site Clinical and Research Infrastructure

1. Does this site have the following facilities in a regular and functioning manner:	
i. Electricity	☐ Yes ☐ No
ii. Backup power supply (generator)	☐ Yes ☐ No
iii. Fresh water supply	☐ Yes ☐ No
iv. Functioning sewerage system	☐ Yes ☐ No
v. Internet access	☐ Yes ☐ No
vi. IT support	☐ Yes ☐ No
vii. Biochemical / clinical laboratory service on site	☐ Yes ☐ No
viii. Blood bank on site	☐ Yes ☐ No
ix. Radiology department on site	☐ Yes ☐ No
x. Antenatal admission<br>If yes, how many beds?	☐ Yes ☐ No
xi. Postnatal admission<br>If yes, how many beds?	☐ Yes ☐ No
xii. Labour ward/room<br>If yes, how many beds?	☐ Yes ☐ No
xiii. Adult intensive care unit on site<br>If yes, how many beds?	☐ Yes ☐ No
xiv. Neonatal intensive care unit / Special Care Newborn Units (SCANU)<br>If yes how many beds?	☐ Yes ☐ No
xv. Family planning unit	☐ Yes ☐ No
2. Does this site have the following equipment available?	
i. Fridge/freezer	☐ Yes ☐ No
ii. Centrifuge	☐ Yes ☐ No
iii. Haemoglobinometer	☐ Yes ☐ No
iv. Obstetric ultrasound	☐ Yes ☐ No
v. Epidural anaesthesia	☐ Yes ☐ No
vi. Gram-staining for bacterial infections	☐ Yes ☐ No
vii. HIV testing	☐ Yes ☐ No
viii. TB screening	☐ Yes ☐ No
3. Does the site have a dedicated research unit?	☐ Yes ☐ No

4. Is there a dedicated, secure space available for research staff, equipment, and storage related to conduct of drug trials?	☐ Yes ☐ No
5. Is there a space at the study site where Investigational Medicine Product (IMP) can be stored securely, and temperature monitored and controlled?	☐ Yes ☐ No
6. Is there a dedicated space at the study site where informed consent can be conducted privately, with a trial participant?	☐ Yes ☐ No
7. Is there a dedicated space and computers for data entry and data management at the study site?	☐ Yes ☐ No

6. Site staffing

1. How many obstetrician-gynaecologists are employed at this site?<br>Is there 24/7 specialist coverage?	☐ Yes ☐ No
2. How many midwives or nurse-midwives are employed at this site?<br>Is there 24/7 specialist coverage?	☐ Yes ☐ No
3. How many consultant neonatologists/paediatricians are employed at this site?<br>Is there 24/7 specialist coverage?	☐ Yes ☐ No
4. How many consultant anaesthesiologists are employed at this site?<br>Is there 24/7 specialist coverage?	☐ Yes ☐ No
5. How many consultant radiologists are employed at this site.<br>Is there 24/7 specialist coverage?	☐ Yes ☐ No
6. How many obstetric care physicians / medical doctors are available at the facility?<br>Is there 24/7 specialist coverage?	☐ Yes ☐ No
7. Is there a pharmacist employed at this site?	☐ Yes ☐ No
8. Can the pharmacy handle drugs/placebo for a double-blinded randomised trial?	☐ Yes ☐ No
9. Are the following research staff employed at this site:	
i. Clinical / research laboratory staff<br>If yes, how many?	☐ Yes ☐ No
ii. Statisticians<br>If yes, how many?	☐ Yes ☐ No
iii. Study data managers<br>If yes, how many?	☐ Yes ☐ No
iv. Research midwives<br>If yes, how many?	☐ Yes ☐ No
v. Research officers<br>If yes, how many?	☐ Yes ☐ No
vi. Supporting administrative staff<br>If yes, how many?	☐ Yes ☐ No

10. Does the site have staff responsible for monitoring site processes and ensuring quality and compliance to GCP guidelines?	☐ Yes ☐ No

7. Site recruitment

1. Does the site have access to a translator for translating study documentation (such as consent forms) into local languages?	☐ Yes ☐ No
2. Is there a current, active relationship with:	
i. Local or regional patient/consumer organisations	☐ Yes ☐ No
ii. National professional association for nurses, midwives, and obstetricians	☐ Yes ☐ No
iii. Local women/mothers' groups	☐ Yes ☐ No
3. Has the site ever stopped a clinical trial due to slow recruitment issues?<br><br>If yes, please describe these issues.	☐ Yes ☐ No

8. Data management

1. Does this site have its own trial data management plan?<br>*: Data management plan documents the processes and procedures employed by organisation to promote consistent, efficient, and effective data management practices for each individual study.*	☐ Yes ☐ No
If yes, is it electronic or paper-based?	
If electronic, what is the system used (e.g., REDCap, OpenClinica)?	
2. Are there policies / SOPs in place for:	
i. Data entry guidelines	☐ Yes ☐ No
ii. Data processing	☐ Yes ☐ No
iii. Ownership of data	☐ Yes ☐ No
iv. Access to data	☐ Yes ☐ No
v. Stewardship of data	☐ Yes ☐ No
2. Are site personnel and investigators trained / able to receive training in clinical	☐ Yes ☐ No

data management practices, including data privacy issues?	

References

Eggleston, A. J., Richards, A., Farrington, E., Tse, W. C., Williams, J., Hewage, A. S., ... & Vogel, J. P. (2022). Randomised trials in maternal and perinatal health in low and middle-income countries from 2010 to 2019: a systematic scoping review. BMJ open, 12(7), e059473.
