# Supplementary material for: Identifying and assessing the capacity and experience of trial sites in low- and middle-income countries for high-quality randomised drug trials in maternal and perinatal health
Source: BMJ Glob Health. 2025 Jul 27;10(7):e018063. doi: 10.1136/bmjgh-2024-018063 (PMC12306368; doi:10.1136/bmjgh-2024-018063)
Supplement: online supplemental appendix 3 [file bmjgh-10-7-s003.pdf]

Interview guide:

Interviewer/s	
Date	
Start and finish time	
Participant name	
Participant site	

Preamble:

Thanks for agreeing to participate in this interview. The purpose of this interview is to complete the maternal clinical trial assessment checklist. This checklist has been developed to assess the potential for trial sites to conduct GCP-compliant clinical trials for novel interventions for maternal/perinatal conditions. For your information, all the comments you provide will be treated as identifiable, and at the completion of the study will be made publicly available in a data repository. Any feedback you provide will be captured through field notes that we will take during this discussion. If there are any questions you do not feel comfortable responding to, you do not have to answer them. You are welcome to ask any questions of us before we start. We would also like to ask your permission to record this interview, so that we may refer back to it if needed. The recording will not be shared outside the research team and will be destroyed at the completion of the study. If you consent to the interview but prefer not to be recorded, we can proceed without the recording.

☐ Verbal consent to participate
☐ Verbal consent to record interview

The aim of this project is to identify and evaluate study sites for conducting high-quality, regulatory Phase III clinical trials of novel medicines for pregnant and postpartum women living in LMICs. To start I can give you a brief overview of what clinical trial networks are: Clinical trial networks are collaborative groups of clinical researchers and trial sites that work together to design and conduct multicentre, randomised controlled trials. There are a number of international clinical trial networks and consortia that have been established for conducting regulatory, GCP-compliant trials on novel medicines for infectious diseases, such as HIV and malaria. While several international consortia exist for clinical trials in maternal and perinatal health, these have largely been for the conduct of non-regulatory, pragmatic trials relating to off-label indications, or testing repurposed medicines.

As part of the Accelerating Innovation for Mothers (AIM) project, we will map clinical trial sites capable of conducting GCP-compliant trials in low- and middle-income countries (LMICs). This project aims to identify and assess the capacity of clinical trial sites in LMICs and establish a new collaboration between those with the ability to conduct high-quality trials for regulatory approval of novel medicines for obstetric complications of pregnancy.

Do you have any questions before we begin the checklist?

If no, or once questions are answered refer to excel spreadsheet and progress through the checklist in numerical order.
