# Supplementary material for: Identifying and assessing the capacity and experience of trial sites in low- and middle-income countries for high-quality randomised drug trials in maternal and perinatal health
Source: BMJ Glob Health. 2025 Jul 27;10(7):e018063. doi: 10.1136/bmjgh-2024-018063 (PMC12306368; doi:10.1136/bmjgh-2024-018063)
Supplement: online supplemental appendix 4 [file bmjgh-10-7-s004.pdf]

## Preamble

Thank you for agreeing to complete this checklist.

The checklist has been developed to assess the potential for trial sites to conduct good clinical practice (GCP)-compliant clinical trials for novel interventions for maternal/perinatal conditions. As part of the Accelerating Innovation for Mothers (AIM) project, we will map clinical trial sites capable of conducting GCP-compliant trials in low- and middle-income countries (LMICs). This project aims to identify and assess the capacity of clinical trial sites in LMICs and establish a new collaboration between those with the ability to conduct high-quality trials for regulatory approval of novel medicines for obstetric complications of pregnancy.

For your information, all the comments you provide will be treated as identifiable, and at the completion of the study **will be made publicly available in a data repository**. If there are any questions you do not feel comfortable responding to, you do not have to answer them. You are welcome to email us

at maureen.makama@burnet.edu.au or annie.mcdougall@burnet.edu.au with any questions you may have.

**Please note that this checklist is to be completed for one site/facility only. If you have multiple sites/facilities in your network, please share the link to the checklist with each site/facility to complete as well.**

By continuing with this survey, you voluntarily agree to participate and provide your responses. Your participation is entirely voluntary, and you have the right to withdraw at any time.

To proceed, please **confirm** your agreement to the information provided being made publicly available

- ◯ Yes, I agree
- ◯ No, I do not agree (exit survey)

Select the date of checklist completion

| | |
|---|---|
| Day | ⌄ |
| Month | ⌄ |

Year 2023

# 1. Identifying information

## Study Site

*Any physical location where a trial is conducted.*

Name:

Address:

City:

State/Province:

Country:

## Primary contact person

Full name:

Job title

Contact number:

Email address:

Affiliation:

Does the primary contact have a valid Good Clinical Practice (GCP) qualification?

## Is the primary contact person the clinical trial unit head or head of department for this site?

- Yes
- No

## Clinical trial unit head or head of department

Full name:

Contact number:

Email address:

Affiliation:

Does the trial unit head have a valid Good Clinical Practice (GCP) qualification?

## Is there a Site Coordinator at this site?

*The person responsible for managing trial activities at this site/facility.*

- Yes
- No

## Site Coordinator

Full name:

Contact number:

Email address:

Affiliation:

Does the site coordinator have a valid Good Clinical Practice (GCP) qualification?

## Principal Investigator (PI)

*The lead researcher responsible for the conduct of a trial at any study site.*

Full name:

Contact number:

Email address:

Affiliation:

Does the PI have a valid Good Clinical Practice (GCP) qualification?

## Are there other Clinical Investigators at this site?

*The person responsible for the conduct of a clinical trial at a trial site.*

Yes

No

How many other **active** Clinical Investigators are at this site?

| | 0 | 1 | 2 | 3 | 4 | 5 | 6 | 7 | 8 | 9 | 10 | 11 | 12 | 13 | 14 | 15 | 16 | 17 | 18 | 19 | 20 | |
|---|---|---|---|---|---|---|---|---|---|---|---|---|---|---|---|---|---|---|---|---|---|---|
| Number of Clinical Investigators | | | | | | | | | | | | | | | | | | | | | | |

Clinical Investigator

Full name:

Contact number:

Email address:

Affiliation:

Does the clinical investigator have a valid Good Clinical Practice (GCP) qualification?

## Clinical Investigator

Full name:

Contact number:

Email address:

Affiliation:

Does the clinical investigator have a valid Good Clinical Practice (GCP) qualification?

## Clinical Investigator

Full name:

Contact number:

Email address:

Affiliation:

Does the clinical investigator have a valid Good Clinical Practice (GCP) qualification?

## Clinical Investigator

Full name:

Contact number:

Email address:

Affiliation:

Does the clinical investigator have a valid Good Clinical Practice (GCP) qualification?

## Clinical Investigator

Full name:

Contact number:

Email address:

Affiliation:

Does the clinical investigator have a valid Good Clinical Practice (GCP) qualification?

## Clinical Investigator

Full name:

Contact number:

Email address:

Affiliation:

Does the clinical investigator have a valid Good Clinical Practice (GCP) qualification?

## Clinical Investigator

Full name:

Contact number:

Email address:

Affiliation:

Does the clinical investigator have a valid Good Clinical Practice (GCP) qualification?

## Clinical Investigator

Full name:

Contact number:

Email address:

Affiliation:

Does the clinical investigator have a valid Good Clinical Practice (GCP) qualification?

## Clinical Investigator

Full name:

Contact number:

Email address:

Affiliation:

Does the clinical investigator have a valid Good Clinical Practice (GCP) qualification?

## Clinical Investigator

Full name:

Contact number:

Email address:

Affiliation:

Does the clinical investigator have a valid Good Clinical Practice (GCP) qualification?

## Please provide the details of any additional Clinical Investigators below

## Is there a finance administration team at this site?

*(The person(s) responsible for maintaining the financial security of a trial site).*

- Yes
- No

## List the contact details of the finance manager for this site

*(The person primarily responsible for maintaining the financial security of a trial site).*

Full name:

Contact number:

Email address: [ ]

Does the site have a secure bank account that allows for international money transfer?

*(capacity for electronic transfer of funds by an international money transfer provider).*

- Yes
- No

## 2. Research experience at the site

Are there any trials in maternal and perinatal health **currently ongoing** at this site?

- Yes
- No

How many trials in maternal and perinatal health are **currently ongoing** at this site?

0 1 2 3 4 5 6 7 8 9 10 11 12 13 14 15 16 17 18 19 20

Number of trials [ ]

Provide the following information for **currently ongoing** maternal and perinatal health trials:

Full name:

Registration number:

Intervention:

GCP Compliant

Trial phase

Is this a regulatory trial (i.e. a trial performed to produce data intended for government or regulatory agency approvals for the sale, use, or manufacture of a relevant product)?

Provide the following information for **currently ongoing** maternal and perinatal health trials:

Full name :

Registration number :

Intervention:

GCP Compliant

Trial phase

Is this a regulatory trial (i.e. a trial performed to produce data intended for government or regulatory agency approvals for the sale, use, or manufacture of a relevant product)?

Provide the following information for **currently ongoing** maternal and perinatal health trials:

Full name :

Registration number :

Intervention:

GCP Compliant

Trial phase

Is this a regulatory trial (i.e. a trial performed to produce data intended for government or regulatory agency approvals for the sale, use, or manufacture of a relevant product)?

Provide the following information for **currently ongoing** maternal and perinatal health trials:

Full name :

Registration number :

Intervention:

GCP Compliant

Trial phase

Is this a regulatory trial (i.e. a trial performed to produce data intended for government or regulatory agency

approvals for the sale, use, or manufacture of a relevant product)?

## Provide the following information for **currently ongoing** maternal and perinatal health trials:

Full name :

Registration number :

Intervention:

GCP Compliant

Trial phase

Is this a regulatory trial (i.e. a trial performed to produce data intended for government or regulatory agency approvals for the sale, use, or manufacture of a relevant product)?

## Provide the following information for **currently ongoing** maternal and perinatal health trials:

Full name :

Registration number :

Intervention:

GCP Compliant

Trial phase

Is this a regulatory trial (i.e. a trial performed to produce data intended for government or regulatory agency approvals for the sale, use, or manufacture of a relevant product)?

Provide the following information for **currently ongoing** maternal and perinatal health trials:

Full name :

Registration number :

Intervention:

GCP Compliant

Trial phase

Is this a regulatory trial (i.e. a trial performed to produce data intended for government or regulatory agency approvals for the sale, use, or manufacture of a relevant product)?

Provide the following information for **currently ongoing** maternal and perinatal health trials:

Full name :

Registration number :

Intervention:

GCP Compliant

Trial phase

Is this a regulatory trial (i.e. a trial performed to produce data intended for government or regulatory agency

approvals for the sale, use, or manufacture of a relevant product)?

Provide the following information for **currently ongoing** maternal and perinatal health trials:

Full name :

Registration number :

Intervention:

GCP Compliant

Trial phase

Is this a regulatory trial (i.e. a trial performed to produce data intended for government or regulatory agency approvals for the sale, use, or manufacture of a relevant product)?

Provide the following information for **currently ongoing** maternal and perinatal health trials:

Full name :

Registration number :

Intervention:

GCP Compliant

Trial phase

Is this a regulatory trial (i.e. a trial performed to produce data intended for government or regulatory agency approvals for the sale, use, or manufacture of a relevant product)?

Please provide details of any other **currently ongoing** maternal and perinatal health trials:

Were there any trials in maternal and perinatal health **completed** at this site in the past five years?

- Yes
- No

How many trials in maternal and perinatal health were **completed** at this site in the past five years?

| | 0 | 1 | 2 | 3 | 4 | 5 | 6 | 7 | 8 | 9 | 10 | 11 | 12 | 13 | 14 | 15 | 16 | 17 | 18 | 19 | 20 | |
|---|---|---|---|---|---|---|---|---|---|---|---|---|---|---|---|---|---|---|---|---|---|---|
| Number of trials | | | | | | | | | | | | | | | | | | | | | | |

Provide the following information for maternal and perinatal health trials **completed** in the past five years:

Full name:

Registration number:

Intervention:

GCP Compliant

Trial phase

Is this a regulatory trial (i.e. a trial performed to produce data intended

for government or regulatory agency approvals for the sale, use, or manufacture of a relevant product)?

Provide the following information for maternal and perinatal health trials **completed** in the past five years:

Full name:

Registration number:

Intervention:

GCP Compliant

Trial phase

Is this a regulatory trial (i.e. a trial performed to produce data intended for government or regulatory agency approvals for the sale, use, or manufacture of a relevant product)?

Provide the following information for maternal and perinatal health trials **completed** in the past five years:

Full name:

Registration number:

Intervention:

GCP Compliant

Trial phase

Is this a regulatory trial (i.e. a trial performed to produce data intended for government or regulatory agency approvals for the sale, use, or manufacture of a relevant product)?

Provide the following information for maternal and perinatal health trials **completed** in the past five years:

Full name:

Registration number:

Intervention:

GCP Compliant

Trial phase

Is this a regulatory trial (i.e. a trial performed to produce data intended for government or regulatory agency approvals for the sale, use, or manufacture of a relevant product)?

Provide the following information for maternal and perinatal health trials **completed** in the past five years:

Full name:

Registration number:

Intervention:

GCP Compliant

Trial phase

Is this a regulatory trial (i.e. a trial performed to produce data intended for government or regulatory agency approvals for the sale, use, or manufacture of a relevant product)?

Provide the following information for maternal and perinatal health trials **completed** in the past five years:

Full name:

Registration number:

Intervention:

GCP Compliant

Trial phase

Is this a regulatory trial (i.e. a trial performed to produce data intended for government or regulatory agency approvals for the sale, use, or manufacture of a relevant product)?

Provide the following information for maternal and perinatal health trials **completed** in the past five years:

Full name:

Registration number:

Intervention:

GCP Compliant

Trial phase

Is this a regulatory trial (i.e. a trial performed to produce data intended for government or regulatory agency approvals for the sale, use, or manufacture of a relevant product)?

Provide the following information for maternal and perinatal health trials **completed** in the past five years:

Full name:

Registration number:

Intervention:

GCP Compliant

Trial phase

Is this a regulatory trial (i.e. a trial performed to produce data intended for government or regulatory agency approvals for the sale, use, or manufacture of a relevant product)?

Provide the following information for maternal and perinatal health trials **completed** in the past five years:

Full name:

Registration number:

Intervention:

GCP Compliant

Trial phase

Is this a regulatory trial (i.e. a trial performed to produce data intended for government or regulatory agency approvals for the sale, use, or manufacture of a relevant product)?

## Provide the following information for maternal and perinatal health trials **completed** in the past five years:

Full name:

Registration number:

Intervention:

GCP Compliant

Trial phase

Is this a regulatory trial (i.e. a trial performed to produce data intended for government or regulatory agency approvals for the sale, use, or manufacture of a relevant product)?

Provide the details for any other maternal and perinatal health trials **completed** in the past five years:

Are there any trials in maternal and perinatal health **planned** (i.e., the protocol is currently being formally considered by an ethics committee) at this site in the next two years?

- Yes
- No

How many trials in maternal and perinatal health are **planned** at this site in the next two years?

0 1 2 3 4 5 6 7 8 9 10 11 12 13 14 15 16 17 18 19 20

Number of trials

Provide the following information for maternal and perinatal health trials **planned** in the next two years:

Full name:

Registration number:

Intervention:

GCP Compliant

Trial phase

Is this a regulatory trial (i.e. a trial performed to produce data intended for government or regulatory agency approvals for the sale, use, or manufacture of a relevant product)?

## Provide the following information for maternal and perinatal health trials **planned** in the next two years:

Full name:

Registration number:

Intervention:

GCP Compliant

Trial phase

Is this a regulatory trial (i.e. a trial performed to produce data intended for government or regulatory agency approvals for the sale, use, or manufacture of a relevant product)?

Provide the following information for maternal and perinatal health trials **planned** in the next two years:

Full name:

Registration number:

Intervention:

GCP Compliant

Trial phase

Is this a regulatory trial (i.e. a trial performed to produce data intended for government or regulatory agency

approvals for the sale, use, or manufacture of a relevant product)?

## Provide the following information for maternal and perinatal health trials **planned** in the next two years:

Full name:

Registration number:

Intervention:

GCP Compliant

Trial phase

Is this a regulatory trial (i.e. a trial performed to produce data intended for government or regulatory agency approvals for the sale, use, or manufacture of a relevant product)?

## Provide the following information for maternal and perinatal health trials **planned** in the next two years:

Full name:

Registration number:

Intervention:

GCP Compliant

Trial phase

Is this a regulatory trial (i.e. a trial performed to produce data intended for government or regulatory agency approvals for the sale, use, or manufacture of a relevant product)?

## Provide the following information for maternal and perinatal health trials **planned** in the next two years:

Full name:

Registration number:

Intervention:

GCP Compliant

Trial phase

Is this a regulatory trial (i.e. a trial performed to produce data intended for government or regulatory agency approvals for the sale, use, or manufacture of a relevant product)?

Provide the following information for maternal and perinatal health trials **planned** in the next two years:

Full name:

Registration number:

Intervention:

GCP Compliant

Trial phase

Is this a regulatory trial (i.e. a trial performed to produce data intended for government or regulatory agency

approvals for the sale, use, or manufacture of a relevant product)?

## Provide the following information for maternal and perinatal health trials **planned** in the next two years:

Full name:

Registration number:

Intervention:

GCP Compliant

Trial phase

Is this a regulatory trial (i.e. a trial performed to produce data intended for government or regulatory agency approvals for the sale, use, or manufacture of a relevant product)?

## Provide the following information for maternal and perinatal health trials **planned** in the next two years:

Full name:

Registration number:

Intervention:

GCP Compliant

Trial phase

Is this a regulatory trial (i.e. a trial performed to produce data intended for government or regulatory agency approvals for the sale, use, or manufacture of a relevant product)?

Provide the following information for maternal and perinatal health trials **planned** in the next two years:

Full name:

Registration number:

Intervention:

GCP Compliant

Trial phase

Is this a regulatory trial (i.e. a trial performed to produce data intended for government or regulatory agency approvals for the sale, use, or manufacture of a relevant product)?

Please provide the details of any other maternal and perinatal health trials **planned** in the next two years:

Has this site previously been part of any international trial networks/collaborations?

- Yes
- No

Provide the name of any international trial networks/collaborations this site has been involved with

Has this site conducted any other GCP compliant trials **outside of maternal and perinatal health** in the past five years?

- Yes
- No

How many other GCP compliant trials **outside of maternal and perinatal health** has this site conducted in the past five years?

0 1 2 3 4 5 6 7 8 9 10 11 12 13 14 15 16 17 18 19 20

Number of trials

Provide the following information for any other GCP-complaint trials **outside of maternal and perinatal health** in the past five years:

Full name:

Registration number:

Intervention:

Population/ trial indication

GCP Compliant

Trial phase

Is this a regulatory trial (i.e. a trial performed to produce data intended for government or regulatory agency approvals for the sale, use, or manufacture of a relevant product)?

## Provide the following information for any other GCP-complaint trials **outside of maternal and perinatal health** in the past five years:

Full name:

Registration number:

Intervention:

Population/ trial indication

GCP Compliant

Trial phase

Is this a regulatory trial (i.e. a trial performed to produce data intended for government or regulatory agency approvals for the sale, use, or manufacture of a relevant product)?

Provide the following information for any other GCP-complaint trials **outside of maternal and perinatal health** in the past five years:

Full name:

Registration number:

Intervention:

Population/ trial indication

GCP Compliant

Trial phase

Is this a regulatory trial (i.e. a trial performed to produce data intended for government or regulatory agency approvals for the sale, use, or manufacture of a relevant product)?

Provide the following information for any other GCP-complaint trials **outside of maternal and perinatal health** in the past five years:

Full name:

Registration number:

Intervention:

Population/ trial indication

GCP Compliant

Trial phase

Is this a regulatory trial (i.e. a trial performed to produce data intended for government or regulatory agency approvals for the sale, use, or manufacture of a relevant product)?

## Provide the following information for any other GCP-complaint trials **outside of maternal and perinatal health** in the past five years:

Full name:

Registration number:

Intervention:

Population/ trial indication

GCP Compliant

Trial phase

Is this a regulatory trial (i.e. a trial performed to produce data intended for government or regulatory agency approvals for the sale, use, or manufacture of a relevant product)?

Provide the following information for any other GCP-complaint trials **outside of maternal and perinatal health** in the past five years:

Full name:

Registration number:

Intervention:

Population/ trial indication

GCP Compliant

Trial phase

Is this a regulatory trial (i.e. a trial performed to produce data intended for government or regulatory agency approvals for the sale, use, or manufacture of a relevant product)?

Provide the following information for any other GCP-complaint trials **outside of maternal and perinatal health** in the past five years:

Full name:

Registration number:

Intervention:

Population/ trial indication

GCP Compliant

Trial phase

Is this a regulatory trial (i.e. a trial performed to produce data intended for government or regulatory agency

approvals for the sale, use, or manufacture of a relevant product)?

## Provide the following information for any other GCP-complaint trials **outside of maternal and perinatal health** in the past five years:

Full name:

Registration number:

Intervention:

Population/ trial indication

GCP Compliant

Trial phase

Is this a regulatory trial (i.e. a trial performed to produce data intended for government or regulatory agency approvals for the sale, use, or manufacture of a relevant product)?

Provide the following information for any other GCP-complaint trials **outside of maternal and perinatal health** in the past five years:

Full name:

Registration number:

Intervention:

Population/ trial indication

GCP Compliant

Trial phase

Is this a regulatory trial (i.e. a trial performed to produce data intended for government or regulatory agency approvals for the sale, use, or manufacture of a relevant product)?

Provide the following information for any other GCP-complaint trials **outside of maternal and perinatal**

**health** in the past five years:

Full name:

Registration number:

Intervention:

Population/ trial indication

GCP Compliant

Trial phase

Is this a regulatory trial (i.e. a trial performed to produce data intended for government or regulatory agency approvals for the sale, use, or manufacture of a relevant product)?

Please provide the details of any other GCP compliant trials outside of maternal and perinatal health

Has this site been monitored by international or external monitors such as a Clinical Research Organization (CRO)?

- Yes
- No

## 3. Ethical and regulatory aspects

Does the site have an ethics committee?

*This is the committee responsible for evaluating the ethical aspects and scientific validity of a proposed study (sometimes known as IRB). In some cases, there may be separate committees for ethics and scientific methods.*

- Yes
- No

How often does the ethics committee meet?

Does the site have internal guidelines (e.g. participant care guidelines) and/or Standard Operating Procedures

(SOPs) related to the following aspects of clinical trial management?

Staff training

Participant recruitment

Clinical trial procedures

Safety management e.g. responding to adverse events (AEs)/serious adverse events (SAEs)

GCP training and compliance

Quality management/ control

In the past five years, has this site been required to stop recruiting to any trial due to a serious safety concern, such as by an ethics review committee of regulatory body?

- Yes
- No

Please describe the circumstances

# 4. Site characteristics

What is the location of the site? (Tick all that apply)

- ☐ Urban: high population and density.
- ☐ Rural: low population and density, centred on agricultural production.
- ☐ Peri urban: mix of rural and urban characteristics

What level of healthcare service is provided by the site? (Tick all that apply)

- ☐ Tertiary: highly specialised medical care.
- ☐ Secondary: specialist healthcare treatment and support.
- ☐ Primary: first point of contact for healthcare.
- ☐ Community
- ☐ Other: ______

What types of care is/are provided at this site: (tick all that apply)

- ☐ Antenatal care: care during pregnancy
- ☐ Labour and childbirth care: care during childbirth
- ☐ Postnatal care: care following childbirth
- ☐ None of the above

How many antenatal visits are performed at this facility each month (on average)?

How many births occur per month (on average, as per most recent year of data)?

How many caesarean sections are performed (on average, as per most recent year of data)?

How many postnatal visits are performed at this facility each month (on average)?

Are maternal and newborn care services located within the same hospital?

- Yes
- No

Which of the following services are available at this hospital:

| | Always | Most of the time | About half the time | Sometimes | Never |
|---|---|---|---|---|---|
| i. Administration of parenteral antibiotics (administration of antibiotics by injection methods) | ○ | ○ | ○ | ○ | ○ |
| ii. Administration of uterotonic drugs (i.e., parenteral oxytocin, tranexamic acid): administration of drugs designed to induce contraction of the uterus | ○ | ○ | ○ | ○ | ○ |
| iii. Administration of magnesium sulphate for pre-eclampsia and eclampsia | ○ | ○ | ○ | ○ | ○ |

| | Always | Most of the time | About half the time | Sometimes | Never |
|---|---|---|---|---|---|
| iv. Manual removal of the placenta | ○ | ○ | ○ | ○ | ○ |
| v. Removal of retained products (e.g., manual vacuum extraction, dilation, and curettage) | ○ | ○ | ○ | ○ | ○ |
| vi. Assisted vaginal birth (e.g., vacuum extraction, forceps) | ○ | ○ | ○ | ○ | ○ |
| vii. Basic neonatal resuscitation (e.g., with bag and mask) | ○ | ○ | ○ | ○ | ○ |
| viii. Caesarean section | ○ | ○ | ○ | ○ | ○ |
| ix. Breech delivery, multiples | ○ | ○ | ○ | ○ | ○ |
| x. Surgery | ○ | ○ | ○ | ○ | ○ |
| xi. Safe abortion (medical and/or surgical) | ○ | ○ | ○ | ○ | ○ |

## Is abortion allowed in the country?

- ○ Yes, until 12 weeks
- ○ [ ] Yes until XX weeks, please specify below:
- ○ Yes, only for medical reasons
- ○ No

Are women with obstetric complications (such as severe pre-eclampsia, peripartum sepsis, postpartum haemorrhage) treated at:

- ○ This site
- ○ Referred to a higher-level facility

## 5. Site Clinical and Research Infrastructure

Does this site have the following facilities in a regular and functioning manner:

| | Always | Most of the time | About half the time | Sometimes | Never |
|---|---|---|---|---|---|
| i. Electricity | ○ | ○ | ○ | ○ | ○ |
| ii. Backup power supply (generator) | ○ | ○ | ○ | ○ | ○ |
| iii. Fresh water supply | ○ | ○ | ○ | ○ | ○ |
| iv. Functioning sewerage system | ○ | ○ | ○ | ○ | ○ |
| v. Internet access | ○ | ○ | ○ | ○ | ○ |
| vi. IT support | ○ | ○ | ○ | ○ | ○ |
| vii. Biochemical/clinical laboratory service on site | ○ | ○ | ○ | ○ | ○ |
| viii. Blood bank on site | ○ | ○ | ○ | ○ | ○ |

| | Always | Most of the time | About half the time | Sometimes | Never |
|---|---|---|---|---|---|
| ix. Radiology department on site | ○ | ○ | ○ | ○ | ○ |
| x. Antenatal Admission. If yes, how many beds? [ ] | ○ | ○ | ○ | ○ | ○ |
| xi. Postnatal Admission. If yes, how many beds? [ ] | ○ | ○ | ○ | ○ | ○ |
| xii. Labour ward/room. If yes, how many beds? [ ] | ○ | ○ | ○ | ○ | ○ |
| xiii. Adult intensive care unit on site. If yes, how many beds? [ ] | ○ | ○ | ○ | ○ | ○ |
| xiv. Neonatal intensive care unit/Special Care Newborn Units (SCANU). If yes, how many beds? [ ] | ○ | ○ | ○ | ○ | ○ |
| xv. Family planning unit | ○ | ○ | ○ | ○ | ○ |

# Does this site have the following equipment available?

| | Most of the time | About half the time | Rarely |
|---|---|---|---|
| i. Fridge/freezer | ○ | ○ | ○ |
| ii. Centrifuge | ○ | ○ | ○ |

| | Most of the time | About half the time | Rarely |
|---|---|---|---|
| iii. Haemoglobinometer | ○ | ○ | ○ |
| iv. Obstetric Ultrasound | ○ | ○ | ○ |
| v. Epidural anaesthesia | ○ | ○ | ○ |
| vi. Gram-staining for bacterial infections | ○ | ○ | ○ |
| vii. HIV testing | ○ | ○ | ○ |
| viii. TB screening | ○ | ○ | ○ |

Does the site have a dedicated research unit?

- ○ Yes
- ○ No

Is there a dedicated, secure space available for research staff, equipment, and storage related to conduct of drug trials?

- ○ Yes
- ○ No

Is there a space at the study site where Investigational Medicine Product (IMP) can be stored securely, and temperature monitored and controlled?

- Yes
- No

Is there a dedicated space at the study site where informed consent can be conducted privately, with a trial participant?

- Yes
- No

Is there a dedicated space and computers for data entry and data management at the study site?

- Yes
- No

## 6. Site Staffing

The following questions are about the staff at the site. For each staff category, please state how many are

employed and whether they have a 24/7 coverage at the site.

How many obstetrician-gynecologists are employed at this site

Is there a 24/7 coverage of obstetrician-gynecologists at this site?

- Yes
- No

How many midwives or nurse-midwives are employed at this site?

Is there a 24/7 coverage of midwives or nurse-midwives at this site?

- Yes
- No

How many consultant neonatologists/pediatricians are employed at this site?

Is there a 24/7 coverage of consultant neonatologists/pediatricians at this site?

- Yes
- No

How many consultant anesthesiologists are employed at this site?

Is there a 24/7 coverage of consultant anesthesiologists at this site?

- Yes

○ No

How many consultant radiologists are employed at this site?

[ ]

Is there a 24/7 coverage of consultant radiologists at this site?

○ Yes

○ No

How many obstetric care physicians/medical doctors are employed at this site?

[ ]

Is there a 24/7 coverage of obstetric care physicians/medical doctors at this site?

○ Yes

○ No

## Is there a pharmacist employed at this site?

- ◯ Yes
- ◯ No

## Can the pharmacy handle drugs/placebo for a double-blinded randomised trial?

- ◯ Yes
- ◯ No

## Which of the following research staff is employed at this site?

*select all that apply and state how many*

- ☐ [ ] Clinical/research laboratory staff
- ☐ [ ] Statisticians
- ☐ [ ] Study data managers
- ☐ [ ] Research midwives
- ☐ [ ] Research nurses

- [ ] Research officers
- [ ] Supporting administrative staff

Does the site have staff responsible for monitoring site processes and ensuring quality and compliance to GCP guidelines?

- Yes
- No

## 7. Site Recruitment

Does the site have access to a translator for translating study documentation (such as consent forms) into local languages?

- Yes
- No

Is there a current, active relationship with:

i. Local or regional patient/consumer organisations. If yes, please list all organisations.

ii. National professional association for nurses, midwives, and obstetricians. If yes, please list all organisations.

iii. Local women/mothers' groups. If yes, please list all organisations.

If applicable, please list any other groups or professional bodies the site engages with to support participant recruitment.

Has the site ever stopped a clinical trial due to slow recruitment issues?

- Yes
- No

Please describe these issues

## 8. Data management

Does this site have its own trial data management plan?

*Data management plan documents the processes and procedures employed by an organisation to promote consistent, efficient, and effective data management practices for each individual study.*

- ○ Yes
- ○ No

Is the data management plan electronic or paper-based?

- ○ Electronic
- ○ Paper-based
- ○ Both

If electronic, what is the system used (e.g., REDCap, OpenClinica)?

## Are there policies/standard operating procedures (SOPs) in place for:

Data entry guidelines

Data processing

Ownership of data

Access to data

Stewardship of data

## Are site personnel and investigators trained or able to receive training in clinical data management practices, including data privacy issues?

- Yes
- No

Powered by Qualtrics
