# Supplementary material for: Identifying and assessing the capacity and experience of trial sites in low- and middle-income countries for high-quality randomised drug trials in maternal and perinatal health
Source: BMJ Glob Health. 2025 Jul 27;10(7):e018063. doi: 10.1136/bmjgh-2024-018063 (PMC12306368; doi:10.1136/bmjgh-2024-018063)
Supplement: online supplemental appendix 5 [file bmjgh-10-7-s005.pdf]

Appendix 5: The current state of sites conducting maternal and perinatal trials in LMICs

To assess the capacity of sites in LMICs that are conducting maternal and perinatal health trials, self-reported data on the capacity of all 49 sites were collected.

Ethical and regulatory aspects

Thirty-seven of the 49 sites (75.5%) reported having an ethics committee, of which most met monthly (21, 56.8%). Thirty-seven (75.5%) sites reported they had been monitored by international or external monitors such as a Clinical Research Organization (CRO). Most sites had internal guidelines and standard operating procedures relating to staff training (42, 85.7%) participant recruitment (40, 81.6%), clinical trial procedures (42, 85.7%), safety management (43, 87.8%), GCP training and compliance (38, 77.6%) and quality management/control (43, 87.8%). None of the sites reported being required to stop recruitment due to safety concerns in the past five years (Table S1).

Table S1. Number of sites with requisite ethical and regulatory aspects for randomised trials (n = 49 sites)

	**Yes n (%)**	**No n (%)**	**No response n (%)**
Ethics committee available	37 (75.5)	12 (24.5)	0
Frequency of meetings of the ethics committee (n = 37)			
Weekly	1 (2.7)		
Fortnightly or twice a month	6 (16.2)		
Monthly	21 (56.8)		
Every 2 months	3 (8.1)		
Quarterly	4 (10.8)		
As required	2 (5.4)		
International or external monitoring done on the site	37 (75.5)	2 (4.1)	10 (20.4)
Internal guidelines and SOPs relating to:			
Staff training	42 (85.7)	5 (10.2)	2 (4.1)
Participant recruitment	40 (81.6)	5 (10.2)	4 (8.2)
Clinical trial procedures	42 (85.7)	4 (8.2)	3 (6.1)
Safety management	43 (87.8)	3 (6.1)	3 (6.1)
GCP training and compliance	38 (77.6)	6 (12.2)	5 (10.2)
Quality management/control	43 (87.8)	4 (8.2)	2 (4.1)
Required to stop recruiting due to safety concerns in the past 5 years	0	49 (100.0)	0

*One site reported meeting either monthly or quarterly depending on the number of submitted proposals, and one site did not report the frequency of meeting

Site facilities and maternal and perinatal health care provision

Most sites (36, 73.5%) were in urban, high population density areas. More than half (26, 53.1%) reported that they provide tertiary level care services, 24 (49%) provided secondary and 12 (24.5%) provided primary level services. Maternal and newborn services were located within the same facility for 96% of sites. Obstetric complications were treated on site by 42 (85.7%) sites and referred to a higher-level facility by seven (14.3%). Antenatal care was provided by 44 (89.8%) sites, labour and childbirth care by 42 (85.7%), postnatal care by 39 (79.6%) and five sites (10.2%) did not provide any of this care (Table S2). The number of antenatal and postnatal visits per month ranged from 100 to <600 for most sites. The number of births and cesarean sections per month also ranged from 100 to <600 for most sites. The number of antenatal, postnatal and neonatal intensive care or special care newborn unit beds ranged from 20 to <50 for most sites. The number of labour ward and adult intensive care unit beds was less than 20 for most sites (Table S2).

Table S2. Site facilities and care provisions (n = 49 sites)

	**Number of sites (%)**
[1]Type of facility	
Peri-urban (mix of rural and urban characteristics)	14 (28.6)
Rural (low population and density, centred on agricultural population)	4 (8.2)
Urban (high population density)	36 (73.5)
[1]Level of healthcare service provided	
Tertiary (highly specialised medical care)	26 (53.1)
Secondary (specialist healthcare treatment and support)	24 (49.0)
Primary (first point of contact for healthcare)	12 (24.5)
Other	3 (6.1)
Maternal and newborn services located within the same facility	47 (95.9)
Obstetric complications treated at:	
This site	42 (85.7)
Referred to a higher-level facility	7 (14.3)
Types of care provided in the facility	
Antenatal care	44 (89.8)
Labour and childbirth care	42 (85.7)
Postnatal care	39 (79.6)
None of the above	5 (10.2)
Number of antenatal visits per month	
<50	10 (20.4)
50 - 99	0
100 - 599	11 (22.4)
600 - 999	9 (18.4)
>1000	8 (16.3)
Missing data	11 (22.4)
Number of postnatal visits per month	
<50	5 (10.2)
50 - 99	3 (6.1)

100 - 599	25 (51.0)
600 - 999	2 (4.1)
>1000	2 (4.1)
Missing data	12 (24.5)
Number of births per month	
<50	0
50 - 99	1 (2.0)
100 - 599	28 (57.1)
600 - 999	9 (18.4)
>1000	5 (10.2)
Missing data	6 (12.2)
Number of cesarean sections per month	
<50	4 (8.2)
50 - 99	12 (24.5)
100 - 599	26 (53.1)
600 - 999	0
>1000	0
Missing data	7 (14.3)
Number of antenatal beds	
<20	13 (26.5)
20 – 49	18 (36.7)
50 – 99	8 (16.3)
≥100	2 (4.1)
Missing data	8 (16.3)
Number of postnatal beds	
<20	12 (24.5)
20 – 49	19 (38.8)
50 – 99	6 (12.2)
≥100	4 (8.2)
Missing data	8 (16.3)
Number of beds in labour ward/room	
<20	33 (67.3)
20 – 49	8 (16.3)
50 – 99	0
≥100	0
Missing data	8 (16.3)
Number of beds in adult intensive care unit	
<20	31 (63.3)
20 – 49	3 (6.1)
50 – 99	2 (4.1)
≥100	0
Missing data	13 (26.5)
Number of beds in neonatal intensive care unit/ special care newborn unit	
<20	18 (36.7)
20 – 49	22 (44.9)
50 – 99	0
≥100	0
Missing data	9 (18.4)

[1]Some sites reported more than one

The following interventions were always available in most sites: administration of parenteral antibiotics, uterotonic drugs, magnesium sulphate for preeclampsia and eclampsia; manual removal of placenta, removal of retained products, assisted vaginal birth, basic neonatal resuscitation, cesarean section, breach and multiple delivery, surgery, safe abortion (medical and/or surgical), and family planning unit.[1] Epidural anesthesia was available in 53% of sites (Table S3). In most sites, abortion was allowed only for medical reasons (28, 57.1%) or up to 28 weeks in others (Table S4). Abortion was not allowed for any reason in two sites (4.1%).

Table S3. Number of sites delivering maternal and newborn health services (n = 49 sites)

	**Yes**<br><br>**n (%)**	**No**<br><br>**n (%)**
Administration of parenteral antibiotics	49 (100.0)	0 (0)
Administration of uterotonic drugs	46 (93.9)	3 (6.1)
Administration of magnesium sulphate for preeclampsia and eclampsia	47 (95.9)	2 (4.1)
Manual removal of the placenta	46 (93.9)	3 (6.1)
Removal of retained products (e.g., manual vacuum extraction, dilation, and curettage)	47 (95.9)	2 (4.1)
Assisted vaginal birth (e.g., vacuum extraction, forceps)	46 (93.9)	2 (4.1)
Basic neonatal resuscitation (e.g., with bag and mask)	47 (95.9)	2 (4.1)
Cesarean section	45 (91.8)	4 (8.1)
Breech delivery, multiples	45 (91.8)	3 (6.1)
Surgery	45 (91.8)	4 (8.1)
Safe abortion (medical and/or surgical)	46 (93.9)	2 (4.1)
Antenatal admissions	41 (83.7)	2 (4.1)
Postnatal admission	41 (83.7)	2 (4.1)
Labour ward/room	38 (77.6)	3 (6.1)
Adult intensive care unit	36 (73.5)	8 (16.3)
Neonatal intensive care unit/ Special care newborn unit	38 (77.6)	4 (8.1)
Family planning unit	41 (83.7)	3 (6.1)
Epidural anesthesia	26 (53.1)	19 (38.8)

[1]This is consistent with most sites offering comprehensive obstetric care.

Table S4. Legality of abortion across countries*

Is abortion allowed?	Countries	Number of sites within specified countries (%)
No	Sierra Leone, Egypt	2 (4.1)
Yes until _ weeks		
12 weeks	Mozambique, South Africa	5 (10.2)
20 weeks	Thailand	2 (4.1)
24 weeks	Colombia, India	5 (10.2)
Yes, on social or economic grounds	Zambia, Rwanda	6 (12.2)
Yes, only for medical reasons (to preserve health or save a person's life)	Botswana, Brazil, Burkina Faso, Democratic Republic of Congo, Nigeria, Pakistan, Tanzania, Uganda, Zimbabwe, Papua New Guinea, Bangladesh, Ghana	29 (59.2)

*Data as validated from the Center for Reproductive Rights website[39]

Site infrastructure

Most sites always had the following facilities functioning regularly: electricity, backup generator, fresh water supply, sewerage system, internet access, Information Technology (IT) support, biochemical/clinical laboratory services, blood bank and radiology department on site (Table S5). The following equipment or tests were available most of the time at >75% of sites: fridge/freezer, centrifuge, hemoglobinometer, obstetric ultrasound, gram-staining for bacterial infections, HIV testing and tuberculosis screening (Table S6).

Table S5. Number of sites with available utilities and services (n = 49 sites)

Facilities	Always n (%)	Most of the time n (%)	About half the time n (%)	Sometimes n (%)	Never n (%)
Electricity	41(83.7)	7 (14.3)	1 (2.0)	0	0
Backup power supply (generator)	44 (89.8)	3 (6.1)	2 (4.1)	0	0
Fresh water supply	43 (87.8)	6 (12.2)	0	0	0
Functioning sewerage system	46 (93.9)	3 (6.1)	0	0	0
Internet access	37 (75.5)	8 (16.3)	1 (2.0)	2 (4.1)	1 (2.0)
IT support	39 (79.6)	6 (12.2)	3 (6.1)	1 (2.0)	0
Biochemical/clinical laboratory service on site	47 (95.9)	1 (2.0)	0	1 (2.0)	0
Blood bank on site	41(83.7)	2 (4.1)	1 (2.0)	0	5 (10.2)
Radiology department on site	39 (79.6)	3 (6.1)	1 (2.0)	0	4 (8.1)

IT, Information Technology

Table S6: Number of sites with available equipment/tests (n = 49 sites)

**Equipment/tests**	**Most of the time n (%)**	**About half the time n (%)**	**Rarely n (%)**
Fridge/freezer	49 (100.0)	0	0
Centrifuge	45 (91.8)	1 (2.0)	3 (6.1)
Hemoglobinometer	46 (93.9)	3 (6.1)	0
Obstetric ultrasound	45 (91.8)	2 (4.1)	2 (4.1)
Gram-staining for bacterial infections	38 (77.6)	4 (8.1)	7 (14.3)
HIV testing	48 (98.0)	0	1 (2.0)
TB screening	44 (89.8)	3 (6.1)	1 (2.0)

Forty-one (83.7%) sites had a dedicated research unit, 44 (89.8%) had a dedicated and secure space for research staff, equipment and storage related to the conduct of drug trials. Investigational Medicine Products (IMPs) could be stored securely, and temperature monitored and controlled in 48 (98%) sites. All sites had a dedicated space where informed consent could be privately obtained and 45 (91.8%) had space and computers for data entry and management (Table S7).

Table S7. Number of sites with available clinical and research infrastructure (n = 49 sites)

	**Yes n (%)**	**No n (%)**	**No response n (%)**
Dedicated research unit	41 (83.7)	8 (16.3)	0
Dedicated and secure space for research staff, equipment, and storage related to the conduct of drug trials	44 (89.8)	5 (10.2)	0
Investigational Medicine Product (IMP) can be stored securely, and temperature monitored and controlled	48 (98.0)	1 (2.0)	0
Dedicated space where informed consent can be obtained privately	49 (100.0)	0	0
Dedicated space and computers for data entry and management	45 (91.8)	4 (8.2)	0

Site staffing

Most sites (>73%) reported having continuous (24 hours/7 days) coverage of obstetrician-gynecologists, nurses or nurse-midwives, neonatologists or pediatricians, anesthesiologists and obstetric care physicians or medical doctors. Only 57% of sites (n = 28) had 24/7 coverage of consultant radiologists. Almost all sites (48, 98%) had a pharmacist who could handle drugs/placebo for a double-blinded trial. In 42 (85.7%) sites, staff were available to monitor progress and ensure quality and compliance with GCP guidelines (Table S8).

Most sites had less than 10 obstetrician-gynecologists (27, 55.1%), neonatologists/pediatricians (29, 59.2%), consultant anesthesiologists (28, 57.1%) and consultant radiologists (33, 67.3%) (Table S8). Nurses or nurse-midwives were 50 or more in 22 (44.9%) sites. Obstetric care physicians or medical doctors were less than 10 in 17 (34.7%) sites and ranged from 10 – 19 in 15 (30.6%) sites. The following research staff were employed by clinical/research laboratory staff (40, 81.6%), research nurses (37, 75.5%), research midwives (30, 61.2%), research officers (33, 67.3%), supporting administrative staff (45, 91.8%), statistician (30, 61.2%) and study data managers (37, 75.5%) (Table S9).

Table S8. Number of sites with clinical staff coverage (n = 49 sites)

**Staffing**	**Yes n (%)**	**No n (%)**	**No response n (%)**
24/7 coverage of obstetrician/gynecologist	39 (79.6)	8 (16.3)	2 (4.1)
24/7 coverage of nurses/nurse-midwives	43 (87.8)	4 (8.2)	2(4.1)
24/7 coverage of neonatologist/ pediatrician	39 (79.6)	9 (18.4)	1 (2.0)
24/7 coverage of consultant anesthesiologists	36 (73.5)	10 (20.4)	3 (6.1)
24/7 coverage of consultant radiologist	28 (57.1)	17 (34.7)	4 (8.2)
24/7 coverage of obstetric care physicians/medical doctor	40 (81.6)	6 (12.2)	3 (6.1)
Is a pharmacist employed?	48 (98.0)	1 (2.0)	0
Can the pharmacists handle drugs/placebo for a double-blinded trial?	48 (98.0)	1 (2.0)	0
Staff available for monitoring progress and ensuring quality and compliance with GCP guidelines	42 (85.7)	7 (14.3)	0

Table S9. Number of sites with clinical and research staff employed (n = 49 sites)

**Staff employed**	**Number of sites (%)**
Obstetrician-gynecologists employed	
None	2 (4.1)
<10	27 (55.1)
10 – 19	7 (14.3)
20 – 29	5 (10.2)
30 – 39	4 (8.1)
40 – 49	1 (2.0)
≥50	1 (2.0)
Nurses and nurse-midwives employed	
None	2 (4.1)
<10	1 (2.0)
10 – 19	6 (12.2)
20 – 29	8 (16.3)
30 – 39	4 (8.1)
40 – 49	3 (6.1)
≥50	22 (44.9)
Neonatologists/pediatricians employed	
None	5 (10.2)
<10	29 (59.2)
10 – 19	10 (20.4)
20 – 29	3 (6.1)
30 – 39	1 (2.0)
40 – 49	0
≥50	0
Consultant anesthesiologists employed	
	8 (16.3)
<10	28 (57.1)
10 – 19	5 (10.2)
20 – 29	4 (8.1)
30 – 39	1 (2.0)
40 – 49	0
≥50	0
Consultant radiologists employed	
None	9 (18.4)
<10	33 (67.3)
10 – 19	3 (6.1)
20 – 29	1 (2.0)
30 – 39	0
40 – 49	0
≥50	0
Obstetric care physicians/medical doctors employed	
None	1 (2.0)
<10	17 (34.7)
10 – 19	15 (30.6)
20 – 29	4 (8.1)
30 – 39	2 (4.1)

40 – 49	2 (4.1)
≥50	5 (10.2)
Research staff employed	
Clinical/research laboratory staff	40 (81.6)
Research nurses	37 (75.5)
Research midwives	30 (61.2)
Research officers	33 (67.3)
Supporting administrative staff	45 (91.8)
Statistician	30 (61.2)
Study data managers	37 (75.5)

Site recruitment

Forty-one (83.7%) sites had access to a translator for consent forms and other study materials. Thirty-nine (79.6%) sites had an active relationship with national professional associations for nurses, midwives and obstetricians, however, only 18 (36.7%) sites had an active relationship with local or regional patient or consumer groups and 15 (30.6%) sites had an active relationship with local women or mothers' groups (Table S10). Only three sites reported having stopped a trial due to slow recruitment. The reasons given were low incidence of the condition among patients visiting the site and challenges of recruitment due to COVID-19.

Table S10. Number of sites with access to recruitment support services (n = 49 sites)

	**Yes n (%)**	**No n (%)**	**No response n (%)**
Access to a translator (e.g., for consent forms)	41 (83.7)	8 (16.3)	0
Current/active relationship with local or regional patient/consumer organizations	18 (36.7)	25 (51.0)	6 (12.2)
Current/active relationship with National professional association for nurses, midwives, and obstetricians	39 (79.6)	7 (14.3)	3 (6.1)
Current/active relationship with local women/mothers' groups	15 (30.6)	28 (57.1)	6 (12.2)
Trials have been stopped due to slow recruitment	3 (6.1)	46 (93.9)	0

Data management

Most sites (36, 73.5%) had a data management plan, of which 22 (44.9%) were both electronic and paper-based, eight (16.3%) were only paper-based and six (12.2%) were only electronic. Most sites had policies and SOPs in place for data entry (37, 75.5%), data processing (33, 67.3%), data ownership (34, 69.4%), access to data (37, 75.5%) and data stewardship (30, 61.2%). In 47 (95.9%) sites, site personnel and investigators were trained or able to be trained in clinical data management practices (Table S11).

Table S11. Number of sites with data management services (n = 49 sites)

	**Yes n (%)**	**No n (%)**	**No response n (%)**
Data management plan	36 (73.5)	13 (26.5)	0
Electronic	6 (12.2)		
Paper-based	8 (16.3)		
Both electronic and paper-based	22 (44.9)		
Policies/SOPs in place for:			
Data entry guidelines	37 (75.5)	9 (18.4)	2 (4.1)
Data processing	33 (67.3)	11 (22.4)	5 (10.2)
Ownership of data	34 (69.4)	11 (22.4)	4 (8.2)
Access to data	37 (75.5)	9 (18.4)	3 (6.1)
Stewardship of data	30 (61.2)	14 (28.6)	5 (10.2)
Site personnel and investigators trained or able to be trained on clinical data management practices (including data privacy issues)	47 (95.9)	1 (2.0)	1 (2.0)

SOPs, Standard Operating Procedures

Forty-three (87.8%) sites reported having a site coordinator, of which 38 (77.66%) had GCP certification. Forty (81.6%) sites reported having principal investigators with GCP certification. Most sites reported having a finance administration team (43, 87.8%), secure bank accounts (47, 95.9%) and previous collaboration with international researchers (45, 91.8%). Overall, most of the sites reported having adequate personnel and infrastructure for the conduct of clinical trials.
